# Enhancing Lipase Immobilization via Physical Adsorption: Advancements in Stability, Reusability, and Industrial Applications for Sustainable Biotechnological Processes

**DOI:** 10.1021/acsomega.4c07088

**Published:** 2024-11-14

**Authors:** Cinthia Silva Almeida, Francisco Simão Neto, Patrick da Silva Sousa, Francisco Izaias da Silva Aires, José Roberto de Matos Filho, Antônio Luthierre Gama Cavalcante, Paulo Gonçalves de Sousa Junior, Rafael Leandro Fernandes Melo, José C. S. dos Santos

**Affiliations:** † Departamento de Engenharia Química, Universidade Federal do Ceará, Campus do Pici, Bloco 709, Fortaleza, CEP 60455760 Ceará, Brazil; ‡ Instituto de Engenharias e Desenvolvimento Sustentável, Universidade da Integração Internacional da Lusofonia Afro-Brasileira, Campus das Auroras, Redenção, CEP 62790970 Ceará, Brazil; § Programa de Pós Graduação em Química, Universidade Federal do Ceará, Campus do Pici, Bloco 940, Fortaleza, CEP 60440-900 Ceará, Brazil; ∥ Departamento de Química Orgânica e Inorgânica, Universidade Federal do Ceará, Fortaleza, 60455-760 Ceará, Brasil; ⊥ Departamento de Engenharia Metalúrgica e de Materiais, Universidade Federal do Ceará−UFC, Campus do Pici, Fortaleza, 60714-903 Ceará, Brazil

## Abstract

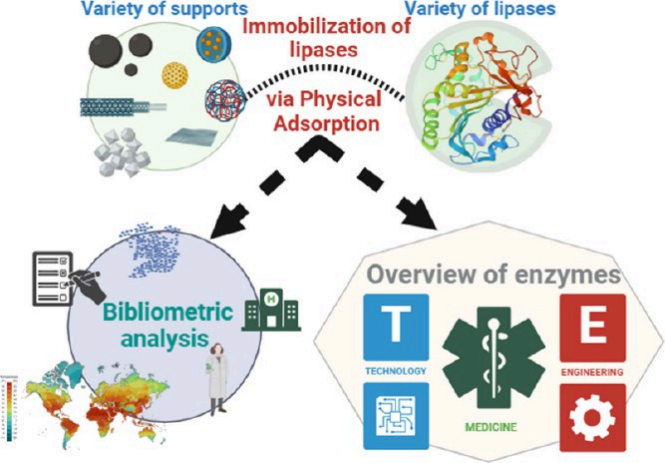

Immobilization of lipases by physical adsorption improves their stability, recovery, and reusability in biotechnological processes. The present review provides an advanced bibliometric analysis and a comprehensive overview of research progress in this field. By searching Web of Science, 39,575 publications were analyzed, and 325 relevant articles were selected. Key journals, countries, institutions, and authors were identified. The most cited articles focus on biofuel production and industrial applications. The analysis revealed four research themes with a focus on the production of biofuel. The physical adsorption method is effective when the appropriate support is used. Despite a decrease in patent applications, industrial interest remains high. Future studies should focus on optimizing support materials and exploring new applications of this technique. The present review provides a detailed understanding of the immobilization of lipases by physical adsorption.

## 1 Introduction

Industries benefit greatly from enzymes, and lipases are particularly noteworthy because of their versatility in catalyzing hydrolytic and synthetic processes. These enzymes exhibit substrate affinity, can function at high substrate concentrations, and are effective in various reaction environments.^1,2^ Lipases can improve product quality by providing excellent stability, solubility, and shelf life, depending on the reactions they are involved in.^3^

**Figure 1:**
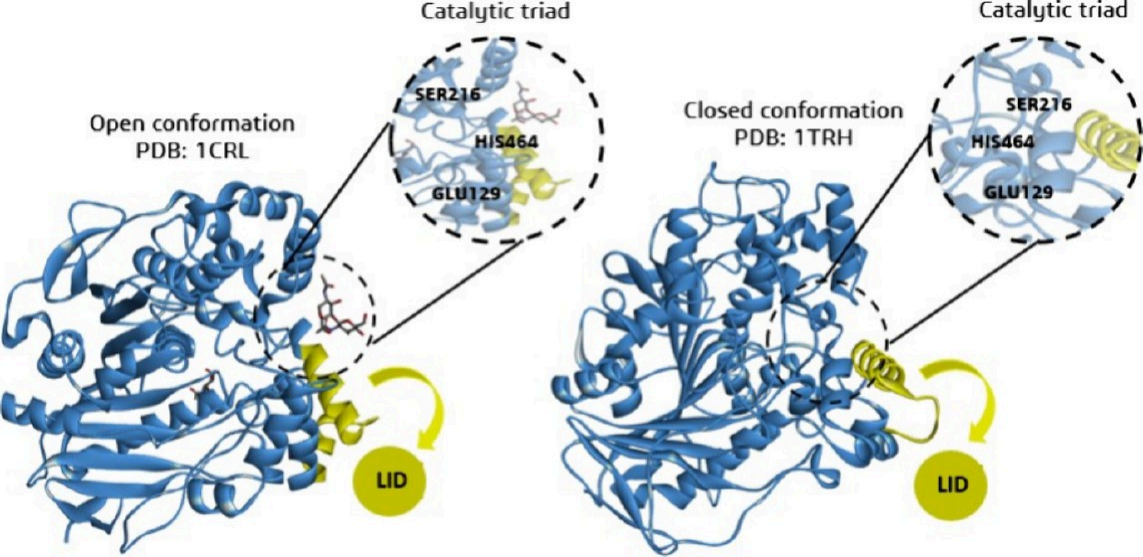
Open and closed conformations of lipase from *Candida rugosa* highlight the enzyme’s catalytic site and the accessibility of ligands to the catalytic triad, showing the preference for the open conformation for ligand–enzyme interaction.

Lipases can catalyze the hydrolysis of insoluble triacylglycerol into glycerol, acylglycerols, and free fatty acids.^4^ They are highly efficient in catalyzing reactions in both aqueous and nonaqueous media because of their stability at extreme temperatures and pH levels and with various solvents.^5^ However, using free-form lipases has limitations such as sensitivity to pH and temperature variations, low operational stability, and difficulty recovering the reaction medium. These issues lead to increased costs and make reuse unfeasible.^6,7^Figure 1 shows the catalytic triad of lipases.

Lipase immobilization is used for enzyme recovery and reuse, allowing direct control of the process.^1,8^ Immobilization methods are based on the chemical and physical interactions between biomolecules and the support matrix.^9^ The most commonly used techniques include physical adsorption (via hydrophobic and van der Waals interactions), chemical adsorption (via covalent and ionic bonds), encapsulation within a matrix or microcapsule, and cross-linking.^10^

**Figure 2:**
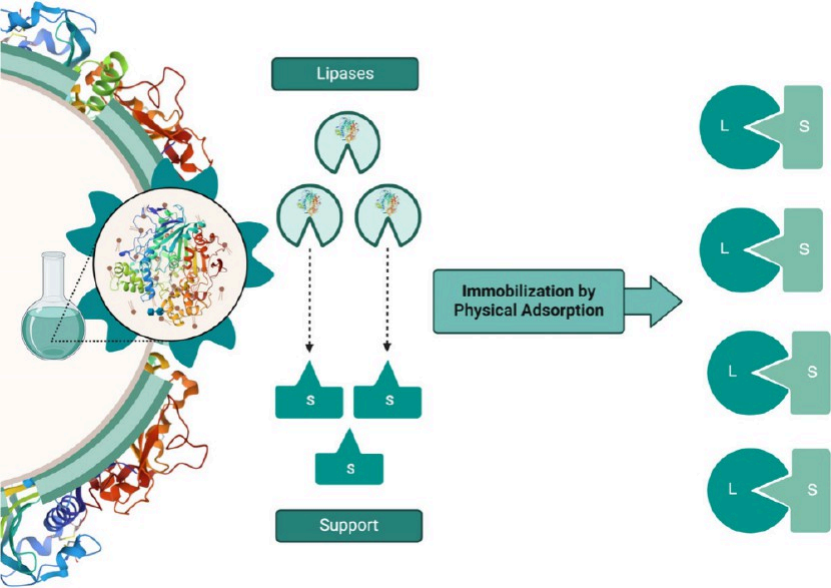
Representation of the lipase enzyme immobilization method using the physical adsorption technique showing the lipase adsorbed on the surface of the support.

Immobilization by physical adsorption involves the enzyme remaining insoluble in the aqueous medium or being retained on the insoluble surface of the support. This method causes a minimal perturbation of the native structure of the enzyme.^11,12^ Compared to other techniques, it is a simple, inexpensive method, does not require support activation, and allows for enzyme reuse. In addition, it can alleviate enzyme inhibition.^13^ However, a disadvantage is that pH variations can lead to enzyme desorption, complicating other process steps, as shown in Figure 2. The effectiveness of this technique depends on many factors, including support surface area, porosity, pore size, enzyme concentration, and the amount of enzyme adsorbed per unit of support.^14,15^

Some analyses that may be performed using advanced bibliometrics include highlighting trends in the growth or decline of research on the topic under study, identifying leading countries and citation patterns, cocreating published articles, and identifying key journals. In addition, we can measure the productivity of authors and coauthors who have published the most.^16^ Bibliometrics is a practical, advanced analytical method that highlights interesting scientific contributions and performs vigorous analyses on the topic.^17,18^

From this point of view, the present study aims to apply advanced bibliometrics to study enzyme immobilization, specifically lipase, using the physical adsorption method to map the knowledge in this field. The justification for this research lies in the need to understand the technique of physical adsorption and to acquire knowledge about enzyme supports for new industrial applications. In addition, it aims to highlight advances in research, current updates and emerging trends in lipase enzyme recovery. Thus, this research aims to contribute to the performance of available technologies and the valorization of biocatalysts.

Therefore, the present review aims to answer the following questions:

- How has the research on lipases immobilized by the physical adsorption method evolved?
- Who are the principal authors of the research on lipase immobilization via physical adsorption?
- What are the main research fields in enzyme immobilization and its applicability?
- What are the primary studies on lipase immobilization that have resulted in efficiency and low cost?

## 2 Methodology

### 2.1 Data Source

Web of Science (WoS) Core Collection (https://www-webofscience.ez11.periodicos.capes.gov.br/wos/woscc/basic-search) (Clarivate, USA) was accessed via the Journals Portal of the Coordenação de Aperfeiçoamento de Pessoal de Nível Superior (CAPES, Brazil).^19,20^ The database was selected because it contains many high-quality scientific citations, which allows for the identification of citations received, the analysis of bibliometric indices, self-citation rates, etc.^21,22^

### 2.2 Data Collection

To reduce bias in the database update, literature on immobilization of lipase enzymes by physical adsorption was consulted on January 20, 2024. The search began with the keywords “lipases,” “immobilization,” and “physical adsorption.” The search strategy was to first enter “lipases” in the search tab, followed by “immobilization” and “physical adsorption.” All terms were searched in English. Years of publication were then defined and set at 2010–2023 to provide a relevant analysis of the last 13 years and to contribute to new investigations. For further analysis, the language was set to English and the file types were limited to journal articles, review articles, and conference articles. All articles retrieved from the Web of Science were downloaded and stored in plain text and image formats.^23,24^

**Figure 3:**
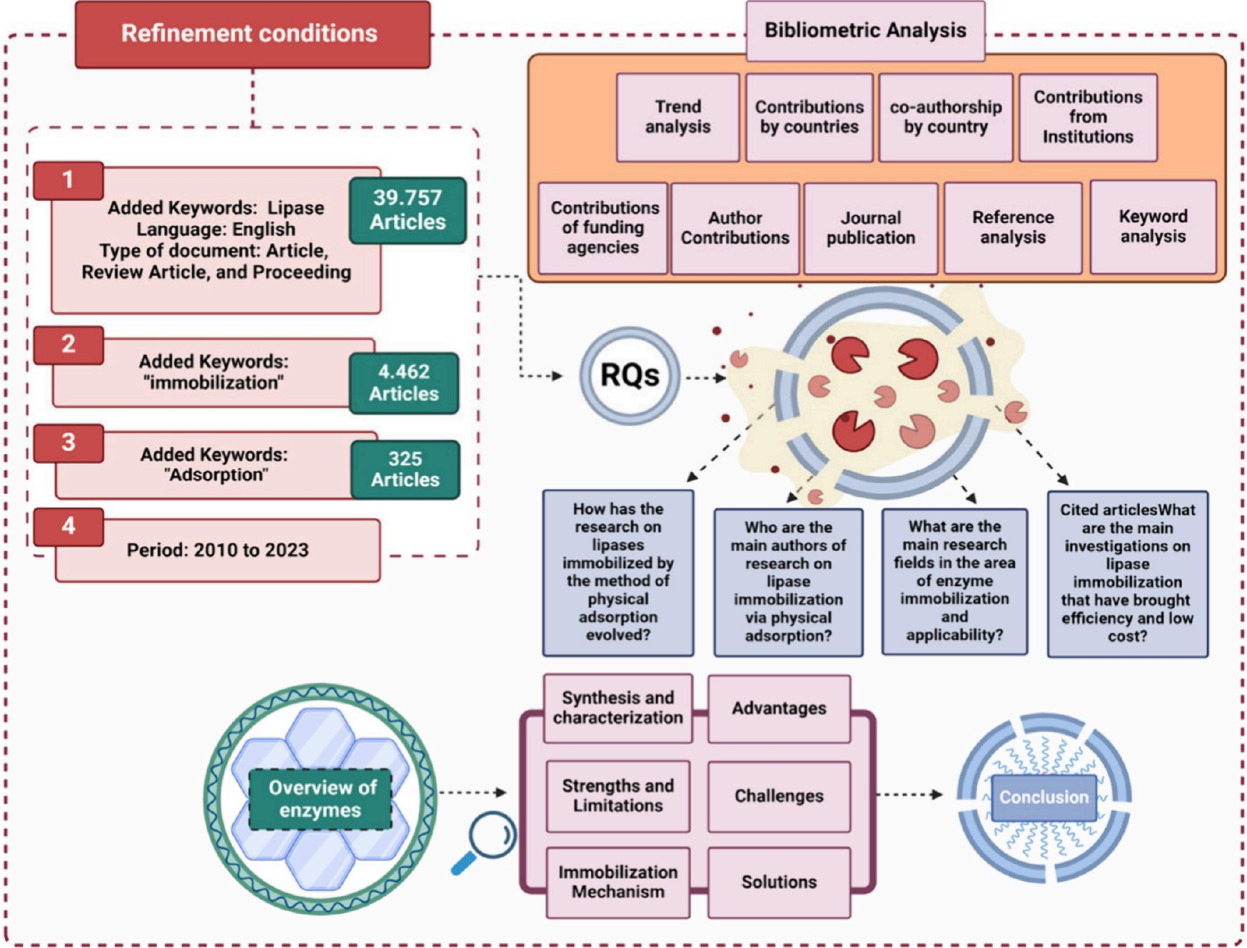
Schematic presentation of the methodology and search criteria for the literature review on the immobilization of lipases by physical adsorption.

Figure 3 illustrates the criteria used to refine the data. The search strategy included using the AND operator for each keyword added and refinement based on the year of publication and the language of the articles. Once the first results were obtained, the article types were selected by limiting the choice to journal articles, review articles and conference articles while excluding other types. The following inclusion criteria were adopted: I) analysis of citations by country/region; II) analysis of citations by institution; III) analysis of coauthorship and cocitation of authors; IV) analysis of cocitation of journals; and V) analysis of co-occurrence of keywords. The present review uses a refined set of bibliometric data to address issues through bibliometric analysis, providing a comprehensive view of the review topic (Figure 3).

### 2.3 Data Extraction

The present review used previous studies for bibliometric analysis.^25−31^ Registers obtained from the CAPES Journals Portal were imported into Microsoft 365 Excel (Microsoft Corporation, USA) for subsequent data visualization. Information extracted from the selected articles included general details such as an annual number of publications, citation frequency, country of origin, authors, journals, affiliated institutions, and funding agencies. Journal performance was assessed using Journal Citation Reports (Clarivate, USA) (available from http://thomsonreuters.com/journalcitationreports/), which provide the Impact Factor (IF) and Quartile (Q1, Q2, Q3, and Q4) of a journal within the field of study. In addition, the *h*-index has been used as a metric to assess scientific productivity, academic status, and research impact at the level of individual researchers, countries, institutions, or journals.^6,7,32^

### 2.4 Data Visualization and Analysis

Data retrieved from the CAPES Journals Portal were displayed by using VOSviewer (version 1.6.17, Leiden University, Netherlands), a Java-based freeware for visualizing bibliometric networks (http://vosviewer.com). VOSviewer allows one to visualize scientific literature from different locations worldwide, highlighting significant countries contributing to the research on a given topic. Network maps in VOSviewer are built based on citation relationships, bibliographic coupling, cocitation or coauthorship, and thus establish connections. Citation analyses are based on the frequency of citations. In contrast, cocitation and co-occurrence analyses refer to the number of times references are cited together and the number of papers in which they co-occur, respectively.^33^ This study used VOSviewer to analyze citations by country/region, institutional citations, author coauthorship and cocitations, journal coauthorship, and keyword co-occurrence.

Network graphs displayed in VOSviewer show intertwined networks representing different parameters, color-coded according to the classification or time of the search. The connections between the networks indicate parameter correlations and are evaluated quantitatively. CiteSpace software (version 5.8.R3, Drexel University, USA) was also used to analyze the data, allowing for greater analytical scope and identifying the authors’ educational institutions, among other details. In addition, Excel was used to create a map of the countries/regions that published the most on the topic and to generate tables and graphs with the information obtained. The results of this research will be made publicly available.

## 3 Results and Discussion

### 3.1 Trend Analysis of Publications and Citations

**Figure 4:**
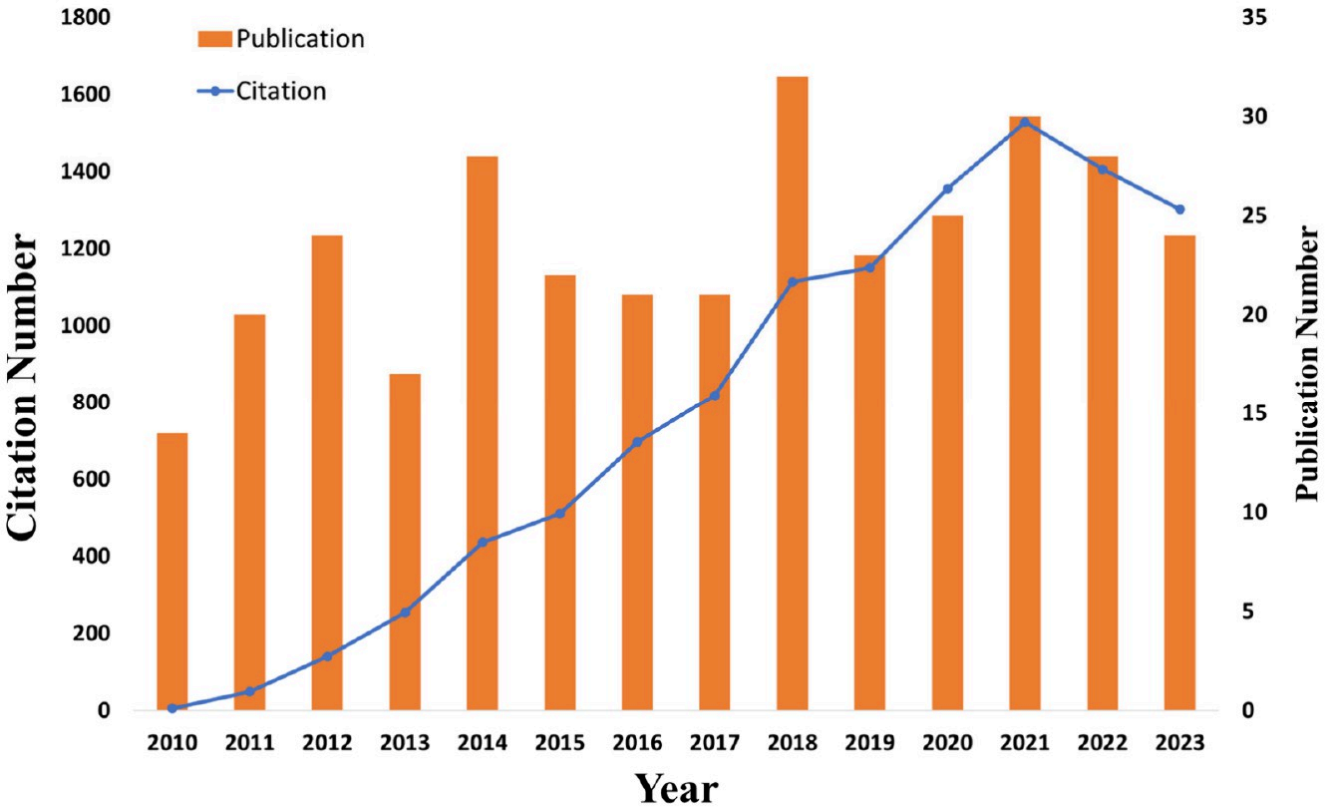
Publications and citations present in the database used in this study, thus related to research on immobilization of lipases by physical adsorption published from 2010 to 2023.

Three hundred and 25 publications were retrieved from WoS after applying filters for keywords, types of study, language, and year of publication (2010–2023), thus limiting the search to the last 13 years. This time frame was chosen to ensure the inclusion of the most recent and relevant publications, allowing for an updated analysis of the topic. Figure 4 shows the distribution of these publications and their citations over the years. There is a global trend in the number of publications and citations related to the immobilization of lipases by physical adsorption, and 2018 had the highest number of published articles, totalling 32. Close behind were 2021 and 2022, with 30 and 28 publications, respectively. In total, these papers accumulated 10,836 citations. However, there is a decrease in the number of publications in 2023. This decrease in 2023 can be attributed to reduced activity or a temporary focus on other research areas. It is typical for the number of publications to grow and fluctuate because of adjustments by journals and periodicals.^34^

One significant aspect of this trend is the cyclical nature of research interest, often influenced by external factors such as funding availability, technological advancements, and global scientific priorities. The slight decline in 2023 may also suggest a saturation point where foundational methods have been well-explored, prompting researchers to pivot toward innovative applications and integrating emerging technologies, such as artificial intelligence and machine learning, to optimize lipase immobilization processes.

Additionally, the high citation count indicates that the work on lipase immobilization by physical adsorption has a substantial scientific impact, contributing valuable knowledge that influences a wide range of subsequent studies. This citation trend underscores the relevance and applicability of these studies in both academic and industrial contexts.

Future research should focus on overcoming current limitations in support materials and exploring novel applications. Advances in material science, such as developing biocompatible and sustainable supports, could significantly enhance the efficiency and cost-effectiveness of lipase immobilization. Moreover, interdisciplinary approaches combining insights from biochemistry, materials science, and computational modeling hold promise for pioneering new frontiers in this domain. This could lead to the developing of highly efficient biocatalysts tailored for specific industrial applications, thereby driving the next wave of innovation in sustainable biotechnological processes.

### 3.2 Contributions by Countries and Regions

The results provided in this section address the first question:

- ***How has the research on lipases immobilized by the physical adsorption method evolved?***

**Figure 5:**
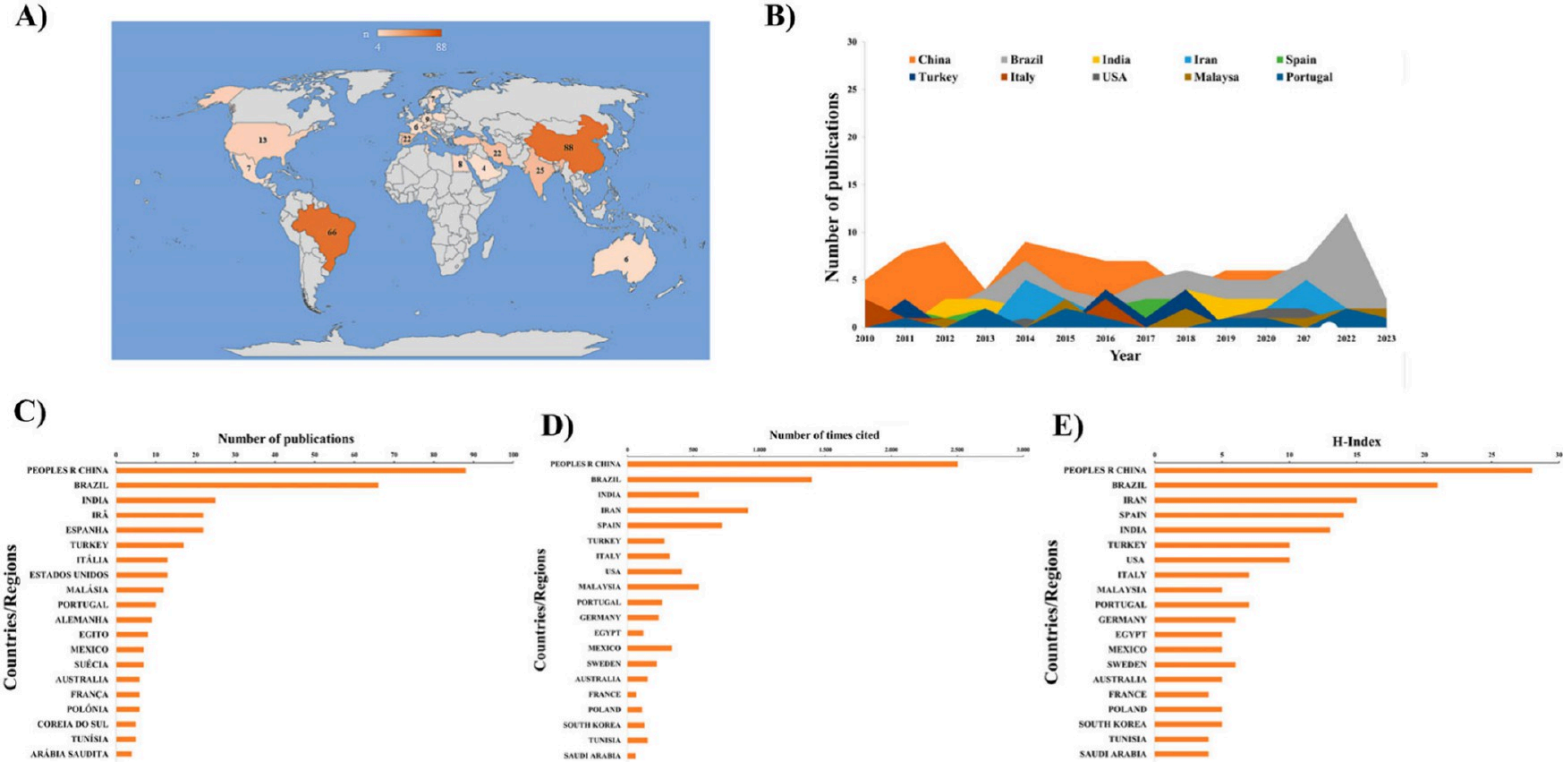
Central countries, institutions, and authors who published between 2010 and 2023. Three hundred twenty-five articles on immobilization by the physical adsorption method. (A) World map showing the countries with the most significant contribution of publications on enzyme immobilization. (B) Trends of publications over the years were based on each country. (C) Several publications are based on the countries/regions that have done the most research on enzyme immobilization by physical adsorption. (D) Number of citations based on the countries/areas, and (E) *h*-index based on the countries with the highest contribution to the publications on enzyme immobilization via physical adsorption.

The world map shown in Figure 5A was generated in Excel after data processing and provides a visualization of the geographic distribution of published papers. It allows the analysis of the publication density in each country through colors and value labels, where darker shades represent a higher number of publications. From 2010 to 2023, Brazil published 66 articles on lipase immobilization via physical adsorption. In addition, the map shows contributions from other countries, with China leading the production with 88 publications on the topic. Thus, Brazil ranks second, just behind China, in the number of publications on this topic. Figure 5B shows the top 10 countries, highlighting the trend in the number of publications, with Brazil peaking in 2022. From 2010 to 2013, China led in the number of publications and continued to do so. Figure 5C provides a quantitative representation of the number of publications by country/region, showing that China ranks first (88 articles; 27%), followed by Brazil (66 articles; 20.3%) and India (25 articles; 7.7%).

Figure 5D illustrates the number of citations of published articles per country/region, providing insight into the citation impact of each country’s work. China leads with 2,502 citations, followed by Brazil with 1,396 and India with 548 citations. Looking at the *h*-index, a measure of productivity and impact, shown in Figure 5E, China, Brazil, and Iran stand out with the highest values. China has an *h*-index of 28, Brazil has an *h*-index of 21, and Iran has an *h*-index of 15. This index quantitatively assesses the performance of authors and the impact of their publications.^35^

In addition, the geographic distribution of research output shown in Figure 4C indicates that Asia, especially China, South America, and Brazil, are major contributors to the field. These regions have shown significant growth in research output in recent years, indicating increasing global interest and investment in enzyme immobilization techniques.

Research’s geographic distribution and impact on lipase immobilization by physical adsorption highlight several critical insights into the field’s evolution and future directions. The prominence of China and Brazil in publication and citation metrics underscores the significant investments these countries have made in biotechnological research and development. This trend reflects broader economic and policy shifts prioritizing sustainable technologies and biobased industrial processes.

The high *h*-index values for China, Brazil, and Iran indicate a high volume of research output and significant impact and recognition within the scientific community. This suggests that the research conducted in these regions is of high quality and relevance, contributing valuable knowledge and advancements to the field of enzyme immobilization. However, the data also reveal disparities in research productivity and impact among different regions, which could be attributed to varying funding levels, infrastructure, and access to cutting-edge technologies. To bridge these gaps, international collaborations and knowledge exchange should be encouraged, leveraging the strengths of leading countries while supporting emerging research hubs.

Future research should focus on developing novel support materials that enhance the efficiency and stability of immobilized lipases. Additionally, exploring the integration of advanced techniques such as nanotechnology, computational modeling, and synthetic biology could unlock new possibilities for optimizing enzyme immobilization. By addressing these challenges and opportunities, the scientific community can drive further innovations in sustainable biotechnological processes, ultimately contributing to global environmental conservation and resource management efforts.

### 3.3 Coauthorship Analysis by Country/Region

**Figure 6:**
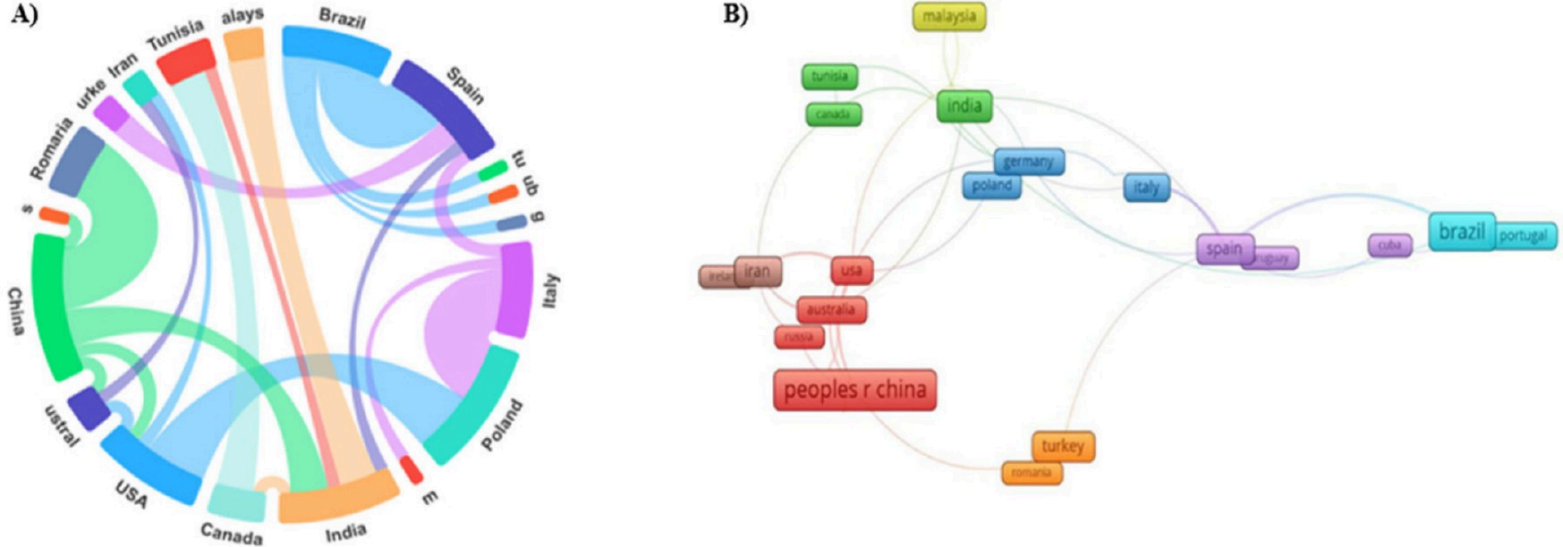
Analysis of the leading countries that collaborated in publishing articles on the immobilization of enzymes by physical adsorption. (A) International collaboration and distribution of countries/regions involved in studying lipase immobilization via physical adsorption. (B) Map of citations of countries/regions generated by VOSviewer. Each node on the map represents a country/region and the size of the number of publications.

Figure 6A illustrates the coauthorship analysis of international collaboration among countries. China played a central role in the research on lipase immobilization by physical adsorption and maintained close cooperation with Brazil, India, Turkey, and Italy. From WoS, 19 countries that contributed the most to the study were selected, each with at least five published documents. These documents were then analyzed using VOSviewer, as shown in Figure 6B. The network map contains approximately 20 nodes and 34 links. The three countries with the highest total link strength (TLS) were Brazil (TLS = 16), Spain (TLS = 15), and the United States (TLS = 13). Each “node” represents the countries that have contributed to the scientific community in recent years, and the larger the node, the more significant the contribution.

The coauthorship analysis reveals that international collaborations significantly bolster scientific advancements in lipase immobilization by physical adsorption. These partnerships not only enhance the quality and impact of the research but also promote the cross-pollination of innovative ideas and techniques. The central role of China in these collaborations highlights the country’s substantial investments in biotechnological research and its commitment to fostering global scientific networks. The strong link strengths of Brazil, Spain, and the United States indicate a strategic alignment of research goals and mutual benefits derived from shared knowledge and resources. This interconnected network of countries suggests a synergistic approach to tackling the complexities of enzyme immobilization, leveraging diverse scientific backgrounds and technological advancements.

Future research should aim to deepen these collaborative efforts by integrating multidisciplinary approaches, combining insights from materials science, biochemistry, and computational modeling to develop more efficient and sustainable immobilization techniques. Additionally, expanding the network to include emerging research hubs in other regions could further enhance the diversity and robustness of the scientific community’s efforts. By fostering a more inclusive and collaborative global research environment, we can accelerate the development of innovative solutions that address current and future challenges in biotechnological processes.

### 3.4 Contributions from Institutions

A total of 10 institutions contributed publications on the topic. Table 1 presents data on the leading universities, of which Universidade Federal de Alfenas led with the highest number of publications (19 articles), followed by Universidade Tiradentes (16 articles) and Consejo Superior de Investigaciones Científicas (CSIC) (15 articles). Brazil ranks fourth out of the top 10 most productive institutions.

**Table 1:** Top 10 Institutions Contributing to Publications on Enzyme Immobilization via Physical Adsorption

rank	institutions	countries/regions	count
first	Universidade Federal de Alfenas	Brasil	19
second	Universidade Tiradentes	Brazil	16
third	Consejo Superior de Investigaciones Cientificas Csic	Spain	15
fourth	Instituto de Catálisis y Petroleoquímica	Spain	13
fifth	Chinese Academy of Sciences	China	12
seventh	Qilu University of Technology	China	9
eighth	Universidade de Aveiro	Portugal	9
ninth	Universidade Federal de Sergipe	Brazil	9
tenth	Egyptian Knowledge Bank Ekb	Tunísia	8

**Figure 7:**
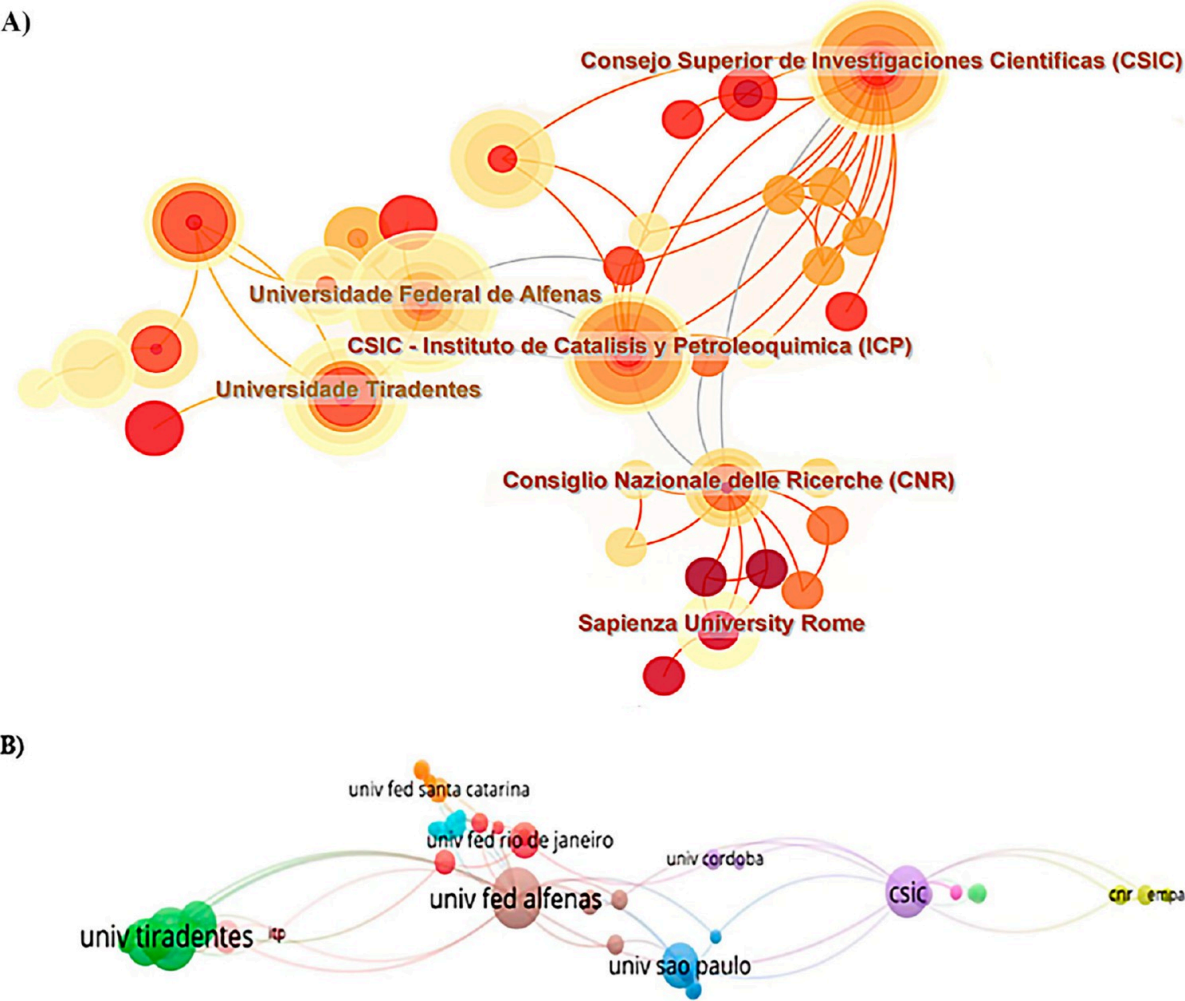
Analysis of the leading institutions that collaborated to publish articles on the topic analyzed. (A) Analysis of the institutions that have published the most on enzyme immobilization (institutional coauthorship) based on CiteSpace. (B) Map of the analysis of citations between institutions identified in the research on immobilization of enzymes by physical adsorption generated by VOSviewer.

For a more detailed analysis of institutional contributions, network maps were generated using CiteSpace and VOSviewer. Figure 7A, generated in CiteSpace, shows the network map of collaborative relationships between institutions. The larger the node, the more significant the contribution of the institution. Universidade Federal de Alfenas, Universidade Tiradentes, and Consejo Superior de Investigaciones Científicas (CSIC) significantly contribute to the research on lipase immobilization by physical adsorption. Figure 7B illustrates the density network map showing the emergence of other universities contributing to the subject under study. Again, the larger the node, the higher the number of publications, with Universidade Federal de Alfenas and Universidade Tiradentes emerging as the central institutions.

The contributions of leading institutions such as Universidade Federal de Alfenas, Universidade Tiradentes, and CSIC underscore the importance of institutional support and investment in advancing biotechnological research. These institutions produce a high volume of publications and serve as centers of excellence that drive scientific progress through collaboration and resource sharing. The network maps reveal the interconnected nature of these research efforts, where partnerships between institutions enhance the overall impact and quality of the research. The prominence of Brazilian institutions in the field highlights the country’s strategic focus on biotechnological innovation and its commitment to addressing global challenges through scientific research. The collaborative relationships depicted in the network maps suggest that these institutions are not working in isolation but are part of a more extensive, synergistic network that includes international partners. This global collaboration is crucial for tackling complex scientific questions and developing scalable solutions.

Future research should further strengthen these institutional collaborations by promoting interdisciplinary projects that leverage the strengths of different research domains. Integrating advanced technologies such as machine learning for data analysis, nanotechnology for improved immobilization supports, and synthetic biology for enzyme engineering can lead to significant breakthroughs. By fostering a culture of innovation and collaboration, institutions can continue to push the boundaries of what is possible in the field of lipase immobilization, ultimately contributing to more sustainable and efficient biotechnological processes.

### 3.5 Contributions of Funding Agencies

Table 2 provides a revealing perspective on the major funding agencies in this area. In particular, China and Brazil emerged as significant contributors, with several agencies from each country appearing on the list. The National Natural Science Foundation of China (NSFC) leads the ranking with 58 contributions (18.18% of the total). This position underscores China’s central role in enzyme immobilization research and development and its commitment to scientific innovation. In Brazil, two agencies stand out as significant contributors: the Conselho Nacional de Desenvolvimento Científico e Tecnológico (CNPq) (48 contributions; 15.04%) and the CAPES (44 contributions; 15.04%). These figures reflect Brazil’s strong commitment to scientific and technological research and highlight the crucial role of these agencies in promoting innovation.

**Table 2:** Leading Funding Agencies on the Topic of Enzyme Immobilization

rank	funding agencies	countries/regions	count	count
1	National Natural Science Foundation of China (Nsfc)	China	58	18.18%
2	Conselho Nacional de Desenvolvimento Científico e Tecnológico (CNPq)	Brazil	48	15.04%
3	Coordenação de Aperfeiçoamento de Pessoal de Nível Superior (CAPES)	Brazil	44	13.79%
4	Fundação de Amparo à Pesquisa do Estado De Minas Gerais (FAPEMIG)	Brazil	14	4.38%
5	Government of Spain	Spain	13	4.07%
6	National Basic Research Program of China	China	12	3.76%
7	Fundação de Amparo à Pesquisa do Estado de São Paulo (FAPESP)	Brazil	10	3.13%
8	Iran National Science Foundation (INSF)	Iran	8	2.50%
9	National High Technology Research and Development Program of China	China	8	2.50%
10	Natural Science Foundation of Shandong Province	China	8	2.50%

Some agencies deserve to be mentioned, namely, Fundação de Amparo à Pesquisa do Estado de Minas Gerais (FAPEMIG), and the Fundação de Amparo à Pesquisa do Estado de São Paulo (FAPESP) in Brazil, the Government of Spain, and the Iran National Science Foundation (INSF) in Iran. These institutions play a fundamental role in funding research projects stimulating discoveries and advances in enzyme immobilization in their regions. This data underscores the importance of international collaboration and adequate funding in advancing enzyme immobilization research and development. Each agency’s contribution drives discoveries and paves the way for advances that may have significant applications in various industries and scientific fields. Therefore, continued investment in these collaborative efforts is critical to driving innovation in this important scientific field.

The significant contributions of funding agencies from China and Brazil highlight the critical role that sustained financial support plays in advancing scientific research. The dominance of the NSFC in China and the prominent roles of CNPq and CAPES in Brazil illustrates how strategic funding can foster high-impact research and technological innovation. This financial backing supports individual research projects, helps build robust research infrastructures, and cultivates skilled scientific communities.

Moreover, the involvement of regional funding bodies such as FAPEMIG and FAPESP in Brazil indicates the importance of localized funding initiatives in addressing specific regional research needs and priorities. These agencies enable the exploration of locally relevant scientific questions while contributing to the global knowledge base. Similarly, the contributions from the Government of Spain and the Iran National Science Foundation (INSF) demonstrate how diverse funding sources can collectively enhance the global research landscape.

The interconnectedness of funding and research output suggests that international collaboration and the sharing of resources are vital for overcoming complex scientific challenges. By pooling expertise and funding from various regions, the scientific community can tackle more significant and more ambitious projects, leading to breakthroughs that might not be possible within a single nation’s resources. Future research should continue emphasizing the integration of advanced interdisciplinary approaches supported by a diversified funding portfolio. This strategy will ensure that enzyme immobilization innovations are sustainable and scalable, maximizing their impact on industrial applications and environmental sustainability. Continued investment in collaborative efforts and funding diversity is essential for maintaining the momentum of scientific discovery and translating research findings into practical, real-world solutions.

### 3.6 Author Contributions

The results provided in this section address the second question:

- ***Who are the principal authors of the research on lipase immobilization via physical adsorption?***

**Figure 8:**
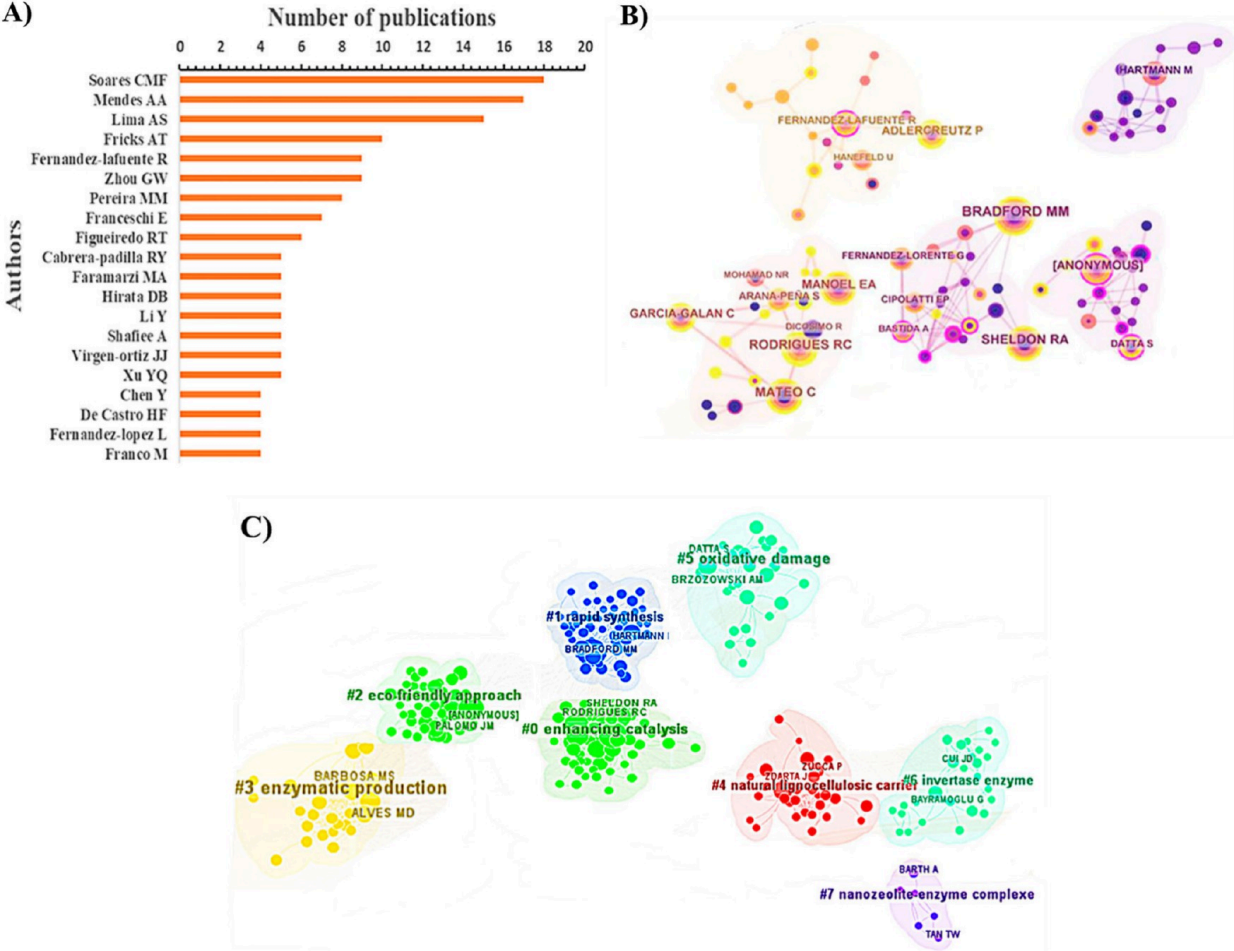
Analysis of the principal authors/coauthors who published articles on the subject between 2010 and 2013. (A) The distribution graph shows the 20 primary authors who have published the most on lipase immobilization via physical adsorption and the number of papers. (B) Coauthorship analysis and graph generated in CiteSpace of the principal coauthors. In the most critical nodes, the number of publications is more unbelievable. (C) Representation generated by CiteSpace with the author’s name and their search categories.

Figure 8A shows the top 20 authors who have published the most on lipase immobilization. The publications of the top 10 authors accounted for 32% of the total literature in this field. Soares CMF (18), Mendes AA (17) and Lima AS (15) were the authors with the highest number of publications, with Soares CMF having the highest number of papers. Figure 8B corresponds to the coauthorship network map, where the node named Bradford MM is the largest, indicating a significant contribution of this coauthor in publications related to the research topic. From a centrality perspective, Bradford MM occupies a central position within the clusters, which are groups of concepts from different issues based on the research area and serve various purposes, such as quantifying the research field.^36^ Other notable authors include Rodrigues RC, Sheldon RA, Mateo C, and Manoel EA. Figure 8C shows the cocitation network among coauthors, where the relevance of authors is determined by the number of times other articles cite their articles. This metric is often used to assess the academic impact of authors. The map also shows clusters representing the research categories of the authors, divided into seven distinct groups: “catalysis improvement” (#0), “rapid synthesis” (#1), “ecological approach” (#2), “enzyme production” (#3), “natural lignocellulosic carrier” (#4), “oxidative damage” (#5), “invertase enzyme” (#6), and “nanozeolite enzyme complex” (#7). These findings have aroused great interest among researchers, focusing on applications to improve catalysis, especially in enzymes.

The author’s contribution analysis highlights the significant role individual researchers and collaborative efforts play in advancing the field of lipase immobilization via physical adsorption. The prominence of authors like Soares CMF, Mendes AA, and Lima AS underscores their extensive contributions and leadership in this area of research. Their prolific output and frequent collaborations have likely facilitated the dissemination of innovative techniques and findings, fostering a richer and more integrated research community.

Bradford MM’s central position in the coauthorship network map indicates a high volume of contributions and a pivotal role in connecting various research efforts and integrating different thematic clusters. This centrality is crucial for cross-pollinating ideas and methodologies, driving the field forward through collaborative synergy.

The cocitation network map reveals the underlying structure of intellectual influence and the formation of research clusters, and each focused on distinct aspects of enzyme immobilization. The presence of diverse clusters such as “catalysis improvement,” “ecological approach,” and “nano zeolite enzyme complex” indicates a multidisciplinary approach to the challenges in this field. This diversity of focus areas suggests that the research community is tackling the problem from multiple angles, incorporating insights from catalysis, environmental science, materials science, and nanotechnology.

Future research should continue to emphasize collaborative efforts, leveraging the strengths of these influential authors and their networks. By fostering deeper interdisciplinary collaborations, researchers can address more complex questions and develop more robust, efficient, and sustainable solutions for enzyme immobilization. Additionally, expanding the analysis to include emerging researchers and institutions can help identify new trends and potential breakthroughs, ensuring the continued evolution and dynamism of the field. This integrated and collaborative approach is essential for translating academic research into practical applications that can significantly impact industrial processes and environmental sustainability.

### 3.7 Journal Publication Analysis

Table 3 shows the journals that have published the most on lipase immobilization. The journal Molecular Catalysis B: Enzymatic published the most articles (17), representing 5.3% of the total publications. The International Journal of Biological Macromolecules and Process Biochemistry rank second (12 publications each; 3.7% of the total). The table also includes an analysis of impact factors and quartile rankings. The International Journal of Biological Macromolecules has the highest impact factor (8.03), followed by Colloids and Surfaces B: Biointerfaces (5.99) and Molecules (4.93). According to the JCR standards for 2022–2023, of the top 10 most active journals, five were classified as Q2 and four as Q1. Data thus integrate the list of journals in the top 25% regarding impact factor and citations.

**Table 3:** Top 10 Journals Published on Enzyme Immobilization via Physical Adsorption, Ranked by Number of Publications

rank	journal title	country	count	percentage (*N*/325) (%)	IF(2022–2023)	quartile in category(2022–2023)	*h*-index
1	Journal of Molecular Catalysis B: Enzymatic	The Netherlands	17	5.329	2.086	Q3	14
2	International Journal of Biological Macromolecules	The Netherlands	12	3.762	8.03	Q1	10
3	Process Biochemistry	United Kingdom	12	3.762	4.88	Q2	10
4	Molecules	Switzerland	11	3.448	4.93	Q1	8
5	Biochemical Engineering Journal	The Netherlands	10	3.135	4.44	Q1	8
6	Bioprocess and Biosystems Engineering	Germany	9	2.821	3.43	Q2	6
7	Colloids and Surfaces B: Biointerfaces	The Netherlands	9	2.821	5.99	Q1	8
8	Applied Biochemistry and Biotechnology	United States	7	2.194	3.09	Q2	6
9	Enzyme and Microbial Technology	United States	7	2.194	3.705	Q2	7
10	Journal of Chemical	United Kingdom	6	1.881	3.70	Q2	4

**Figure 9:**
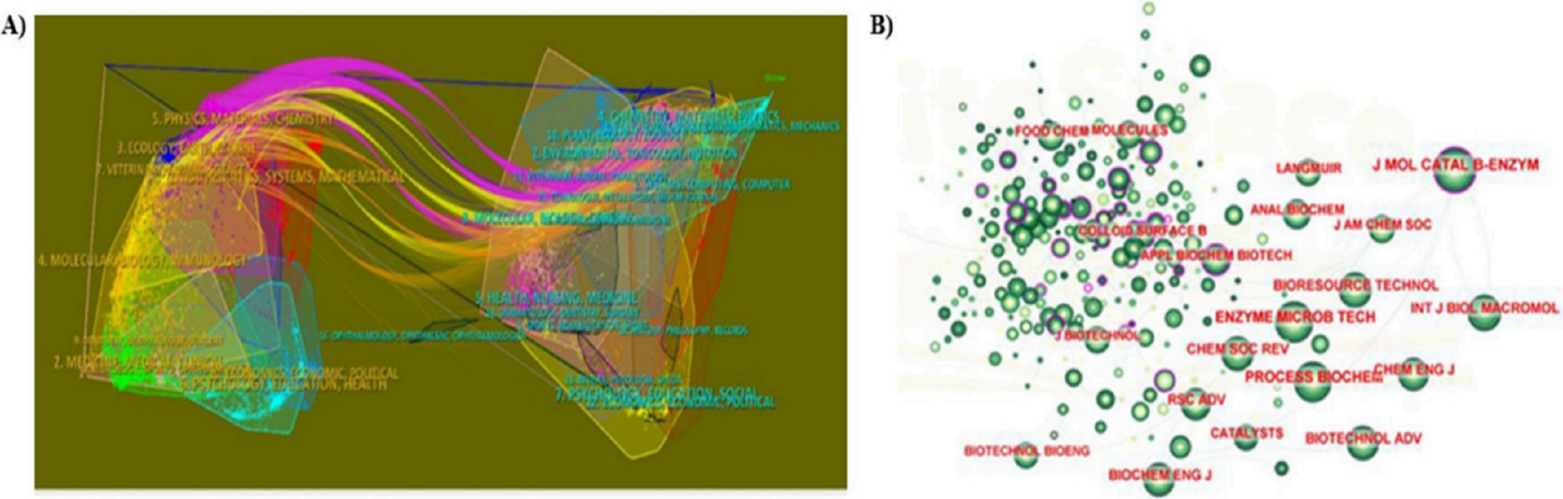
Analysis of the prominent journals that published articles on the subject between 2010 and 2013. (A) Overlay map of journals generated in CiteSpace. Representation of different research topics in lipase immobilization using the physical adsorption method. (B) Analysis of journal cocitation. The size of the nodes represents the number of times the journal was cited.

In addition, Figure 9A overlaps two maps, revealing the general trends of the scientific portfolio in a single visualization. The results showed that the published studies were mainly directed to journals in three categories: (I) Physics, Materials, and Chemistry; (II) Molecular Biology and Immunology; and (III) Medicine, Medical, and Clinical. Figure 9B shows the cocitation map of journals, with the *Journal of Molecular Catalysis B: Enzymatic* (*J. Mol. Catal. B- Enzyme*) having the highest centrality, followed by *Enzyme and Microbial Technology* (*Enzyme Microb. Tech.*). Based on these analyses, it is likely that future research developments in this area will also be published in the journals listed in Table 3.

The analysis of journal publications reveals essential insights into the dissemination and impact of research on lipase immobilization via physical adsorption. High-impact journals in the top quartiles indicate that the research is prolific, of high quality, and relevant. The clustering of publications in journals related to physics, materials, and chemistry suggests that advancements in this field are closely tied to material science innovations and chemical engineering processes.

The high impact factor of the International Journal of Biological Macromolecules highlights the growing interest in the biochemical and molecular aspects of enzyme immobilization. This trend points to a deeper exploration of the fundamental mechanisms underlying the immobilization process and its effects on enzyme activity and stability.

The cocitation map further illustrates the central role of specific journals in shaping the research landscape. Journals like Molecular Catalysis B: Enzymatic and Enzyme and Microbial Technology serve as hubs for high-impact research, facilitating the exchange of ideas and driving forward the field’s frontiers. This network of high-impact journals ensures that significant discoveries and advancements reach a broad audience, fostering further research and innovation.

Future research should aim to maintain and expand publication in these high-impact journals, leveraging their platforms to enhance visibility and impact. Emphasizing interdisciplinary approaches and collaborations will be key to addressing complex challenges in enzyme immobilization. Researchers can develop more efficient and sustainable immobilization techniques by integrating insights from material science, molecular biology, and chemical engineering. Continued focus on publishing in top-tier journals will help ensure that the latest advancements are widely disseminated and adopted, driving academic research and industrial applications forward.

### 3.8 Reference Analysis

The results provided in this section address the third question:

- ***What are the main research fields in enzyme immobilization and its applicability?***

**Figure 10:**
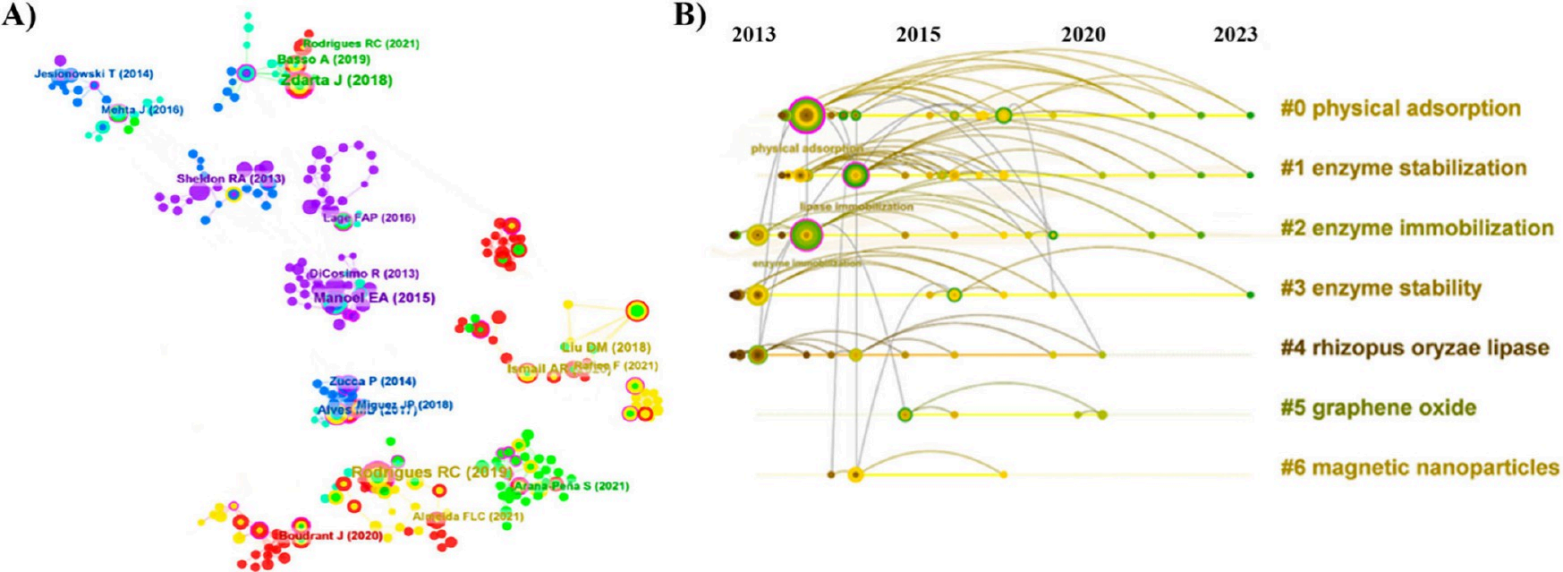
Analysis of the references highlighted in the database used for bibliometric analysis. (A) CiteSpace-generated representation of authors and year of publication of papers. (B) Visualization of the cocitation reference timeline and cluster representation describing seven significant themes, labeled and color-coded.

Table 4 lists the most influential articles on enzyme immobilization, using citation counts as a proxy for influence and relevance. This tabular representation is a valuable resource for researchers to identify areas of focus and trends in lipase immobilization by physical adsorption. Data includes article title, year of publication, first author, and number of citations received, providing a comprehensive view of each article’s impact on the scientific community. Table 4 shows the top 10 most cited articles published between 2010 and 2023. The most cited article, with 1,917 citations, is by Roger A. Sheldon, titled “Enzyme Immobilization in Biocatalysis: Why, What and How”,^13^ published in Chemical Society Reviews in 2013. In second place is the article by Mohamad Nur Royhaila, titled “An Overview of Technologies for Immobilization of Enzymes and Surface Analy”,^37^ published in *Biotechnology & Biotechnological Equipment* in 2015, totaling 431 citations. Figure 10A shows a network map illustrating the relationships in the cocitation network of references with author names and publication years, providing a temporal view of the field of study. Figure 10B highlights the characteristics of emerging and future research topics. The most relevant cluster identified is “physical adsorption” (#0), followed by “enzyme stabilization” (#1) and “enzyme immobilization” (#2). In addition, clusters were observed, indicating the use of specific materials such as magnetic nanoparticles and graphene oxide. These materials are often studied as supports for lipase enzymes, suggesting a trend of studies focusing on these materials.

**Table 4:** Ten Most Cited Articles on Enzyme Immobilization via Physical Adsorption

title	journal	first author	year	citations	references
Enzyme immobilization in biocatalysis: why, what and how.	Chemical Society Reviews	Sheldon, Roger A.	2013	1917	(13)
An overview of technologies for immobilization of enzymes and surface analysis techniques for immobilized enzymes.	Biotechnology & Biotechnological Equipment	Mohamad, Nur Royhaila	2015	431	(37)
Metal–organic frameworks and inorganic nanoflowers: a type of emerging inorganic crystal nanocarrier for enzyme immobilization	Catalysis Science & Technology	Wu, Xiaoling	2015	212	(38)
Graphene oxide immobilized enzymes show high thermal and solvent stability.	Nanoscale	Hermanova, Sona	2015	176	(39)
Immobilization of cellulase enzyme on superparamagnetic nanoparticles and determination of its activity and stability.	Chemical Engineering Journal	Khoshnevisan, Kamyar	2011	171	(40)
Immobilization of enzyme biocatalyst on natural halloysite nanotubes.	Catalysis Communications	Zhai, Rui	2010	166	(41)
Effect of protein load on stability of immobilized enzymes.	Enzime And Microbial Technology	Fernandez- Lopez, Laura	2017	164	(42)
Magnetic-metal organic framework (magnetic- MOF): A novel platform for enzyme immobilization and nanozyme applications.	International Journal of Biological Macromolecules	Nadar, Shamraja S.	2018	145	(43)
Covalent immobilization of lipase onto aminopropyl-functionalized hydroxyapatite- encapsulated-gamma-Fe2O3 nanoparticles: A magnetic biocatalyst for interesterification of soybean oil.	Food Chemistry	Xie, Wenlei	2017	136	(44)
Enzymatic transesterification for biodiesel production from used cooking oil, a review.	Journal of Cleaner Production	Moazeni, Faegheh	2019	132	(45)

The analysis of references reveals critical thematic areas and emerging trends within the field of enzyme immobilization. The high citation counts of foundational articles indicate their profound impact on guiding subsequent research directions. The focus on physical adsorption, enzyme stabilization, and exploring novel support materials like magnetic nanoparticles and graphene oxide suggests a dynamic and evolving research landscape.

Physical adsorption remains a prominent method due to its simplicity and effectiveness. However, recent studies are increasingly exploring hybrid approaches that combine physical adsorption with other immobilization techniques to enhance enzyme activity and stability. Integrating magnetic nanoparticles and graphene oxide as support materials represents a significant advancement, offering unique properties such as high surface area, ease of recovery, and functional versatility. These materials improve immobilization efficiency and open new avenues for biocatalysis in nonaqueous media and under harsh operational conditions.

Future research should optimize these novel materials and hybrid techniques to achieve higher enzyme loadings, enhanced stability, and better reusability. Additionally, understanding the molecular interactions between enzymes and support materials at a deeper level can provide insights into designing more effective immobilization strategies. Advanced characterization techniques, such as atomic force microscopy and molecular dynamics simulations, could play a crucial role in elucidating these interactions.

Furthermore, interdisciplinary collaborations integrating insights from materials science, nanotechnology, and molecular biology will be essential to drive innovation. By fostering a holistic approach, researchers can develop next-generation immobilization techniques that improve biocatalytic processes and contribute to sustainable industrial practices. Such advancements will be pivotal in addressing global energy, environment, and health challenges, demonstrating the far-reaching impact of research in enzyme immobilization.

### 3.9 Analysis of Keywords

The results provided in this section address the fourth question:

- ***What are the primary studies on lipase immobilization that have resulted in efficiency and low cost?***

Keyword co-occurrence analysis is crucial in bibliometric studies as it highlights the most discussed topics and future challenges researchers may face. This analysis reveals the specific words used in a given research area. To analyze the number of words related to lipase immobilization by physical adsorption, the Total Link Strength (TLS) metric was used. TLS is an indicator to quantitatively assess the closeness of collaborations, with high TLS values indicating strong collaborations between authors, institutions, and countries. The highest TLS value means the most robust collaboration. Table 5 analyses the most frequent keywords based on the values obtained using the VOSviewer software. Among the top 20 keywords, the most frequent were “lipase” (140 occurrences), “immobilization” (136 occurrences), and “adsorption” (120 occurrences). This shows the consistency of the topic under study, as even the less frequently cited words are closely related to the research topic.

**Table 5:** Twenty Keywords Most Frequently Used in the Search Analysis

rank	keywords	no. of occurrences	TLS^a^	rank	keywords	no. of occurrences	TLS^a^
**1**	Lipase	140	845	**11**	Covalent immobilization	34	254
**2**	Immobilization	136	815	**12**	Hydrolysis	39	254
**3**	Adsorption	120	797	**13**	Nanoparticles	38	248
**4**	Enzyme immobilization	88	609	**14**	Sílica	33	239
**5**	Stability	78	559	**15**	Esterification	31	232
**6**	*Candida rugosa* lipase	51	356	**16**	Biocatalyst	30	220
**7**	Mesoporous sílica	44	316	**17**	Biocatalysis	33	218
**8**	Physicaladsorption	41	291	**18**	Support	27	203
**9**	Purification	37	279	**19**	Chitosan	28	194
**10**	Enzymes	38	258	**20**	Stabilization	28	190

a TLS: total link strength.

**Figure 11:**
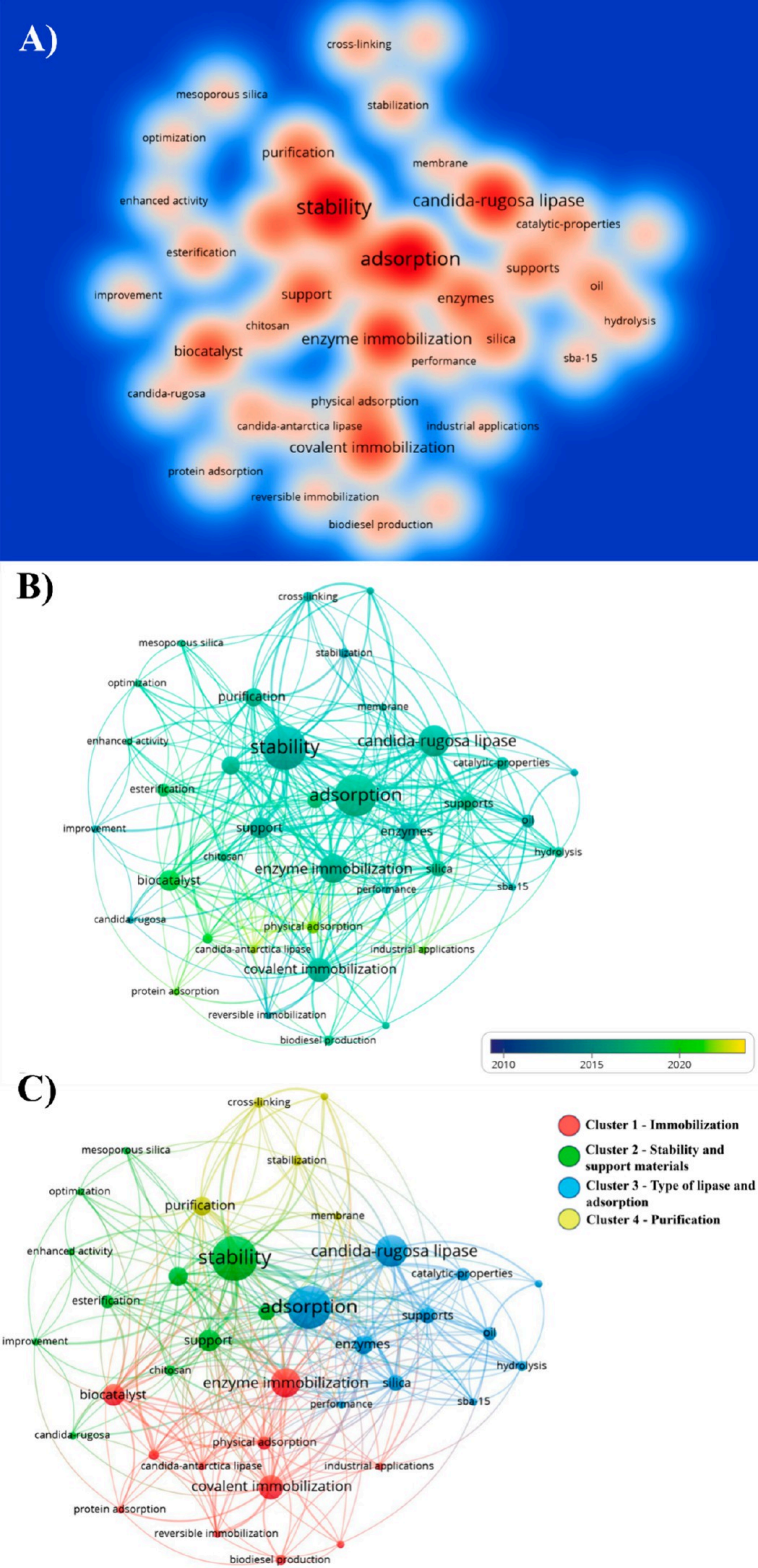
Analysis of the main keywords highlighted within the database used in the bibliometric analysis. (A) Keyword density generated by VOSviewer. It shows that the deeper the node’s color, the more frequently the keywords appear. (B) Co-occurrence analysis over the years. The dark blue nodes in 2010 and around 2015 are the nodes with light green tones—the period with the highest frequency of words—and the yellow node highlights the topic of physical adsorption. (C) The representation of keyword co-occurrences was obtained from VOSviewer, which highlighted the main clusters. Red color, cluster 1 on immobilization; cluster 2 (green) on enzyme stability and support materials. Cluster 3 (blue) refers to the types of lipases and the adsorption method, and cluster 4 (yellow) to the purification process.

Network maps and density maps were generated in VOSviewer to provide a more detailed analysis of the keyword data. Figure 11A shows the keyword density map, which allows for observing different words associated with the research topic. Figure 11B shows the association between all the keywords, with more intense colors indicating a more significant number of catalogued words. From 2010 to 2015, the color intensity was predominantly green, showing a homogeneous distribution. Figure 11C shows that all identified keywords can be divided into four groups: “lipase,” “immobilization,” “adsorption,” and “carrier materials.” These four clusters represent the main research directions in lipase immobilization. In addition, the data showed that newer keywords such as “lipase in particular (*Candida antarctica* and *Candida rugosa*)”, “immobilization materials”, and “biodiesel production and industrial application” may become primary research points in the coming years. These groups highlight the most critical issues in the research of lipase immobilization via physical adsorption.

(I) **Lipases:** Lipases are the most widely used enzymes in the industrial sector, including but not limited to food, cosmetics, pharmaceuticals, agrochemicals, and biodiesel production.^46,47^ In this context, the application of lipases in biofuel production has gained relevance because of their ability to catalyze feedstocks with high acidity and moisture, which facilitates the utilization of waste oils (e.g., frying oils).^48^ This provides an alternative for industrial-scale biodiesel production using a feedstock that would otherwise be discarded and potentially cause environmental damage.
  The lipase enzymes identified through bibliometric analysis, *Candida antarctica* and *Candida rugosa* are fascinating. *Candida rugosa* can hydrolyze the triacylglycerols ester bonds and catalyze transesterification and esterification reactions, showing a broad and efficient substrate specificity.^49^*Candida antarctica* produces two different lipases: lipase A (CALA A) and lipase B (CALA B). CALA A is stable at acidic pH and has an isoelectric point of 7.5.^50^ The immobilized CALA B lipase can be adsorbed on hydrophobic surfaces when used with hydrophobic supports.^51,52^ Studying these enzymes is essential because of their numerous potential applications.^53^
(II) **Immobilization materials:** Immobilization of enzymes on support materials that can coat the enzyme and enhance its efficacy has attracted considerable interest. Various support materials have been studied for this purpose, including polymer matrices,^54^ porous materials, carbon nanotubes,^55^ membranes,^56^ magnetic materials,^57^ and agroindustrial residues (e.g., cashew bagasse).^58^ Further studies with other residues are attractive because of their low-cost, sustainable alternatives. Similarly, the use of magnetic nanoparticles and graphene oxide, as highlighted in the bibliometric analysis, is noteworthy.^59^
  Besides immobilization materials, techniques to make the process efficient and cost-effective are also essential. Immobilized lipase on cotton cloth, a residue from the textile industry, using the physical adsorption method.^60^ She concluded that it was a simple, economical method that achieved efficiency in hydrolysis and ester synthesis reactions.^61^ This demonstrates the versatility of this technique and adds value to textile industry waste.
(III) **Application - Biosensor:** A biosensor is an analytical device capable of combining a biological element with a physicochemical component.^62,63^ The chemical information reveals the concentration of the analyte and transforms it into a signal recognized by another system.^64^ This highlights the importance of this study and its relevance for future research, as it represents an innovation for various fields of knowledge. An example is medicine, where biosensors effectively screen cancer.^65,66^

The keywords analysis reveals the multifaceted nature of research on lipase immobilization via physical adsorption, highlighting critical areas that have achieved efficiency and cost-effectiveness. The frequent occurrence of terms such as “lipase,″ “immobilization,″ and “adsorption” underscores the core focus of the research community on optimizing these processes. Identifying specific enzymes like Candida antarctica and *Candida rugosa* reflects targeted efforts to explore and exploit their unique catalytic properties, which have significant industrial implications.

Exploring various immobilization materials, ranging from traditional polymer matrices to advanced nanomaterials like graphene oxide, signifies a robust and ongoing search for optimal supports that enhance enzyme activity and stability. This diversity in materials research is pivotal for developing tailored solutions that can address specific industrial needs, from biofuel production to pharmaceuticals.

Moreover, applying lipase immobilization in biosensors represents a frontier that bridges enzymology with cutting-edge analytical technology. The potential of biosensors in medical diagnostics, environmental monitoring, and industrial quality control highlights the broad applicability and transformative impact of immobilized enzymes.

Future research should continue integrating these diverse themes, fostering innovations combining enzyme specificity with advanced material science. Emphasizing interdisciplinary approaches and leveraging computational modeling to predict and enhance enzyme-support interactions can lead to breakthroughs in immobilization efficiency. Such efforts will be crucial in driving sustainable industrial processes and expanding the utility of immobilized lipases across various sectors, ultimately contributing to a more sustainable and technologically advanced society.

## 4 Overview of Enzyme Preparation via Physical Adsorption

### 4.1 Synthesis and Characterization of Biocatalyst

**Figure 12:**
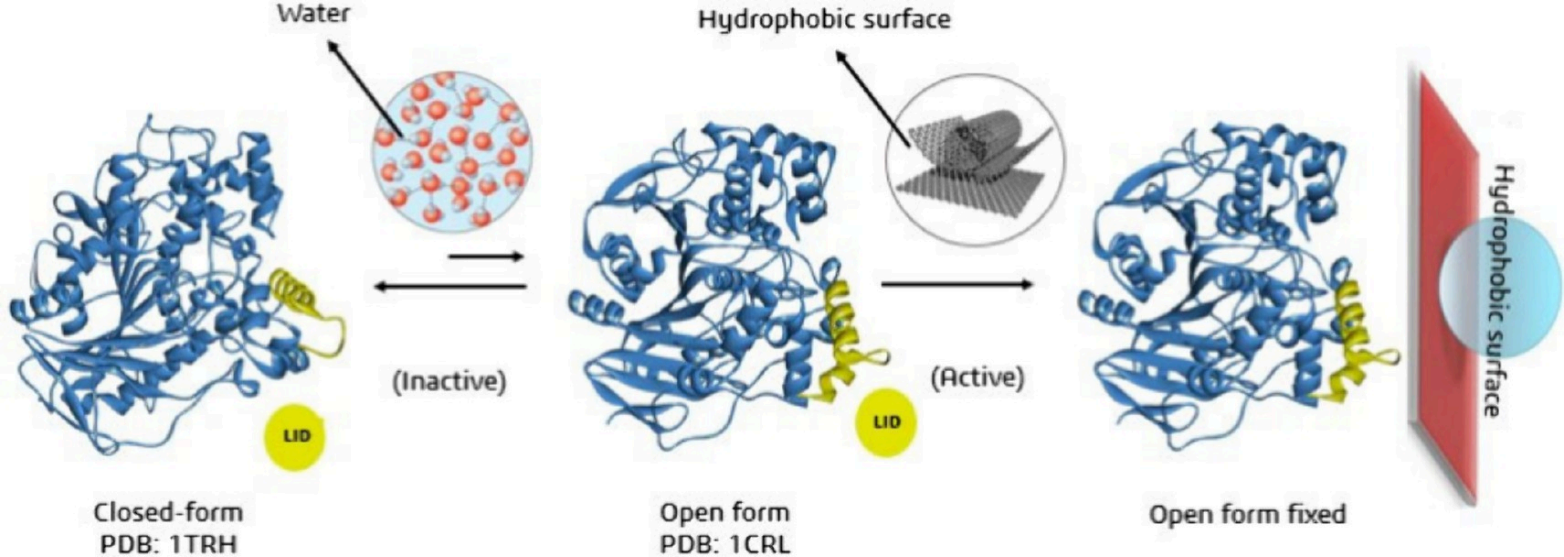
Interaction mechanism of lipase from *Candida rugosa* via interaction with hydrophobic surfaces showing the enzyme’s different open and closed conformations.

The synthesis of biocatalysts is constantly evolving, driven by the industry’s growing interest in improving its products and processes. Therefore, developing efficient, controlled, low-cost and reproducible methods is a crucial research field.^67,68^ A support material is initially obtained for synthesizing an immobilized biocatalyst, which is then used to immobilize the enzyme of interest. There are numerous support materials available; however, for their selection, several physical and chemical properties must be analyzed, such as pH, morphology, resistance to microbiological attack, hydrophobic and hydrophilic characteristics, porosity, nonporosity of the material, among others.^69−71^Figure 12 shows a representation of a lipase interacting with a hydrophobic surface in its open or closed conformation, which should also be considered in lipase immobilization.

Supports can be divided into either organic or inorganic categories.^72,73^ Organic supports are divided into natural and synthetic polymers, including chitosan, alginate, starch, and agarose.^74,75^ Natural polymeric organic supports offer easy degradation, low cost, abundant availability, no environmental impact, high thermal resistance, and easily activated chemical structures.^76^ Synthetic polymeric organic carriers provide a variety of physical forms and chemical structures that can be combined to form the support.^77−80^

On the other hand, inorganic supports offer high mechanical strength, resistance to organic solvents, easy recovery, and thermal stability, and do not undergo structural modifications under varying pH, temperature, and pressure.^81^ Examples of materials that comprise this support group are silica gel, alumina, metal oxides, and zirconia.^82−84^

Once the support material has been selected, enzyme immobilization methods for synthesis are also investigated, which can be categorized into chemical and physical processes. Physical techniques include physical adsorption, which is simple, reversible, and can occur through hydrophobic interactions, hydrogen bonding, and van der Waals forces.^85,86^ Its advantages include immobilization under mild conditions without significant structural changes to the biomolecule.^87^ However, a disadvantage is the possibility of enzyme desorption because of temperature, pH, and agitation variations, which require careful process control.^88,89^ Nevertheless, due to their hydrophobic properties, low ionic strength, and enzyme stabilization, physical adsorption is the most commonly used method for lipases.

Pacheco et al. (2020) stated that in characterizing lipases immobilized by physical adsorption, they found optimal temperature and pH values of 41 °C and 7.75, respectively, resulting in an average enzymatic activity of 158.4 U/g. They compared this with the free form of the enzyme and found that at a temperature of 41 °C, the immobilized biocatalyst retained 70.19% of its initial activity, while the free enzyme retained 56.71%.^90^ This demonstrates the efficiency of the physical adsorption method in immobilizing lipase enzymes.

The focus on lipase immobilization via physical adsorption highlights several vital aspects that are pivotal to advancing this technique. Current methodologies predominantly emphasize selecting appropriate support materials and optimizing adsorption conditions to enhance enzyme stability and activity. While physical adsorption offers advantages such as simplicity and mild immobilization conditions, it is not without its challenges. One significant drawback is enzyme desorption under varying operational conditions, which can compromise the biocatalyst’s effectiveness and reuse.

Advancements in this field have increasingly focused on developing new support materials with enhanced interaction capabilities. For instance, the incorporation of magnetic nanoparticles and graphene oxide improves immobilization efficiency and facilitates easy recovery and reuse of the biocatalysts. However, these advanced materials come with higher costs and more complex preparation processes, which may limit their widespread application in industrial settings.

Moreover, critical analysis of current methodologies reveals a need better to understand the molecular interactions between lipases and support surfaces. Techniques such as molecular dynamics simulations and atomic force microscopy could provide deeper insights into these interactions, enabling the design of more robust and effective immobilization strategies. Additionally, addressing the issue of enzyme desorption requires innovative approaches, such as covalent modification or the development of hybrid immobilization techniques that combine the benefits of physical adsorption with stronger binding methods.

While significant progress has been made in lipase immobilization by physical adsorption, ongoing research must continue to tackle these challenges. Enhancing immobilized lipases’ stability, reusability, and cost-effectiveness will be critical for their broader adoption in industrial applications, ranging from biodiesel production to pharmaceuticals. By integrating multidisciplinary approaches and leveraging advanced material science, researchers can push the boundaries of what is possible with enzyme immobilization, paving the way for more sustainable and efficient biocatalytic processes.

#### 4.1.1 Applications of Biocatalysts

Particular note is the ability of lipases to stabilize under different operating conditions and to recognize a wide range of substrates, as they can catalyze triglyceride hydrolysis as well as esterification, transesterification, aminolysis, and lactonization reactions.^91,92^ These properties allow their application in various industrial sectors, including the food, pharmaceutical, and biofuel industries.^93−95^

Aromatic esters are essential in the pharmaceutical and food sectors and are widely used because of their various properties.^96^ These compounds can be obtained by fermentative processes or directly extracted from natural products.^97^ However, these methods often have low productivity, high costs, and require extensive purification steps, making them less attractive for industrial applications.^96,98,99^ Enzymatic extraction of these compounds has emerged as an exciting alternative because of its low energy consumption and high productivity.^100,101^

Applications of biocatalysts combined with artificial intelligence (AI) have revolutionized several industries, offering improved efficiency and precision in processes ranging from pharmaceuticals to environmental remediation.^102,103^ Using AI algorithms, biocatalysts can be optimized for specific reactions, enhancing their activity and selectivity.^104,105^ In pharmaceuticals, AI-assisted biocatalysis enables rapid screening and design of enzymes for drug synthesis, reducing development time and costs.^106,107^ Similarly, in industrial biotechnology, AI can analyze and predict highly complex conditions such as temperature and pH to maximize product yield and minimize waste.^108−110^ In addition, AI-driven biocatalysis is increasingly used in environmental applications such as wastewater treatment and bioremediation, where enzymes are engineered to effectively degrade pollutants.^111−113^ By integrating AI with biocatalysts, industries are achieving higher sustainability and resource efficiency levels, leading to significant advances in green chemistry and bioprocessing.^114^

**Figure 13:**
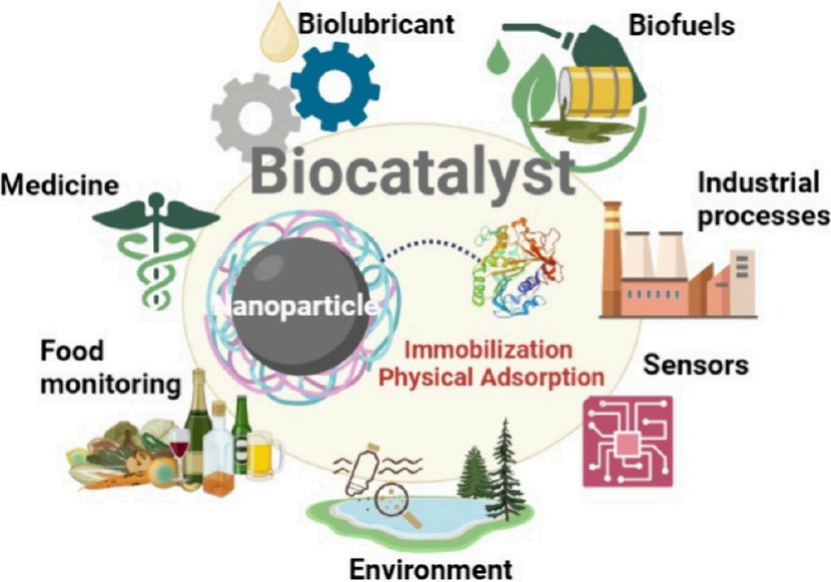
Schematic representation of the various possible applications for lipases immobilized by physical adsorption was found in the database and collected for the bibliometric analysis.

Figure 13 illustrates some of the main applications of immobilized lipases using various nanoparticle options through physical adsorption immobilization.

Focusing specifically on the immobilization of lipase by physical adsorption, this technique presents a blend of simplicity and effectiveness, making it highly attractive for industrial applications. Current methodologies in physical adsorption leverage hydrophobic interactions, hydrogen bonding, and van der Waals forces to attach lipase molecules onto support materials. However, the main challenge remains the potential desorption of enzymes under fluctuating operational conditions such as changes in pH, temperature, and agitation.

Critically analyzing the methodologies, it is evident that while traditional supports like silica gel and activated carbon are adequate, there is a burgeoning interest in using advanced materials such as magnetic nanoparticles and graphene oxide. These materials offer enhanced surface areas and unique interaction capabilities, significantly improving enzyme loading and stability. However, preparing these advanced supports can be complex and costly, posing a barrier to widespread adoption.

Recent advancements have shown promising results in hybrid approaches, combining physical adsorption with covalent binding techniques to mitigate enzyme desorption issues. This hybrid strategy retains the advantages of mild immobilization conditions while providing a stronger attachment to the support, thus enhancing the operational stability of the immobilized enzymes.

Moreover, integrating computational tools and AI can further refine the immobilization process. By predicting optimal immobilization conditions and tailoring support materials at the molecular level, researchers can achieve higher efficiency and robustness in enzyme immobilization. Such interdisciplinary approaches are crucial for overcoming existing limitations and driving innovation in the field.

Future research should focus on the scalable synthesis of advanced support materials and developing cost-effective hybrid immobilization techniques. Additionally, understanding the molecular dynamics of enzyme-support interactions through advanced characterization methods will provide deeper insights into designing more effective immobilization strategies. By addressing these challenges and leveraging technological advancements, the immobilization of lipases by physical adsorption can be optimized for broader industrial applications, contributing significantly to sustainable biocatalytic processes.

#### 4.1.2 Reactors Used in Biocatalysts

The enzyme reactor or bioreactor interacts with the enzyme and the substrate to form the products and is widely used in various processes.^115−117^ The choice of reactor is influenced by factors such as operating characteristics, immobilization support format, substrate type, catalyst surface, etc.^111,118−120^

For triglyceride synthesis, reactors include stirred tank reactors (STR) operated in batch mode and fixed bed reactors (FBR) operated in total recycle or continuous mode. Of these two types, enzymatic reactions are mainly performed in FBRs.^90,121,122^ FBRs contain a cylindrical internal column filled with biocatalysts, where the substrate in liquid or gaseous form flows upward or downward within the reactor.^123^

The advantages of a fixed-bed reactor include ease of operation, low-cost, high product yield, low shear stress, and ease of biocatalyst recovery since it remains fixed, making it suitable for industrial-scale use.^124,125^ However, disadvantages include potential bed plugging, high-pressure drop in fluid flow through the bed, and limitations in mass and heat transfer.^126^

Focusing specifically on the immobilization of lipase by physical adsorption within bioreactors, several critical factors influence this technique’s efficiency and practicality. Current methodologies predominantly utilize FBRs due to their ability to maintain a fixed position for biocatalysts, facilitating easy recovery and reuse. However, one of the main challenges in using FBRs is the potential for bed plugging and high-pressure drops, which can significantly hinder the operational efficiency and scalability of the process.

Advancements in reactor design have aimed to address these challenges by optimizing the flow dynamics within the reactor. For instance, incorporating advanced materials such as magnetic nanoparticles in the immobilization process can enhance the uniformity of the biocatalyst distribution within the reactor, thereby reducing the likelihood of bed plugging and improving mass and heat transfer rates. Additionally, computational fluid dynamics (CFD) simulations have been instrumental in designing reactors with improved flow characteristics and reduced pressure drops.

Furthermore, integrating hybrid immobilization techniques within these reactors can enhance the stability and activity of the immobilized lipases. By combining physical adsorption with covalent binding or entrapment methods, it is possible to achieve a more robust attachment of the enzymes to the support material, thereby reducing the risk of desorption and extending the operational lifespan of the biocatalysts.

Future research should focus on developing scalable and cost-effective reactor designs that incorporate these advanced materials and hybrid immobilization techniques. Moreover, a deeper understanding of the molecular interactions between the immobilized enzymes and the support materials within the reactor environment can provide valuable insights for optimizing reaction conditions and improving overall process efficiency. By addressing these challenges and leveraging technological advancements, the immobilization of lipases by physical adsorption within bioreactors can be further optimized for broader industrial applications, contributing significantly to sustainable biocatalytic processes.

#### 4.1.3 Related Patents

In the literature, analysis of lipase immobilization via physical adsorption has shown a decrease. However, much work remains to be done. Issues such as enzyme stability and economic feasibility have gained prominence as they are inherent aspects of the study.

At the academic level, research on lipase immobilization by physical adsorption significantly impacts biofuel applications and is attracting great interest in the industrial sector. A search for patents on robust platforms or databases, such as the United States Patent and Trademark Office (USPTO) and the European Patent Office (EPO), revealed 671 international patents related to this topic from 1980 to December 2023.

**Figure 14:**
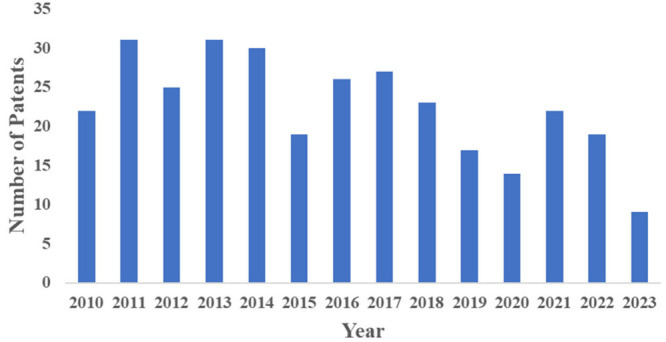
Evolution of patents filed with the United States Patent and Trademark Office (USPTO) and the European Patent Office (EPO) between 2010 and 2023 covering the immobilization of lipases using the physical adsorption method.

The volumes of patents granted or filed in recent years have shown that industries represent more than 59.6% of the patents filed or granted, while academic institutions hold about 40.4%. This percentage of industries can be attributed to the extensive use of these enzymes in the sector and a representation of their benefits for processes. Figure 14 reveals a graph illustrating the significant increase in patent volumes over the past years.

Some countries lead in the number of patents related to enzyme immobilization by physical adsorption. The United States, Japan, Australia, Canada, and China are at the top ranking. Furthermore, considering that English is the predominant language in the scientific community, it is relevant to mention that virtually all readings are available in English. Although there has been a decrease in the number of patents in the field of lipase immobilization by physical adsorption, the study remains promising for obtaining new immobilization materials. When combined with this method, these materials can potentially improve processes.

The analysis of patents related to lipase immobilization via physical adsorption provides critical insights into the evolving landscape of industrial and academic research. Despite a noted decrease in literature publications, the robust patent activity underscores this technology’s ongoing innovation and industrial application. The high proportion of patents filed by industries reflects immobilized lipases’ practical significance and commercial viability, particularly in sectors such as biofuels, pharmaceuticals, and food processing.

Current methodologies in patent filings reveal a persistent focus on enhancing enzyme stability and economic feasibility. These patents often detail advanced materials and hybrid immobilization techniques to overcome traditional challenges associated with enzyme desorption and operational longevity. For instance, patents frequently cite using novel support materials like magnetic nanoparticles and graphene oxide, which offer superior binding properties and ease of enzyme recovery.

A critical analysis of these methodologies highlights both advancements and persistent challenges. While novel materials and hybrid techniques have shown promise, scalability, cost-effectiveness, and process integration remain significant hurdles. Furthermore, the variability in enzyme performance due to fluctuating operational conditions necessitates the development of more robust immobilization strategies.

Future research and patent activity should address these challenges by focusing on scalable and economically viable solutions. Innovations in material science, particularly developing biocompatible and sustainable supports, could play a pivotal role. Additionally, integrating computational modeling and AI-driven optimization can streamline the design of immobilization processes, enhancing their efficiency and applicability across diverse industrial contexts.

By aligning academic research with industrial needs and fostering interdisciplinary collaborations, the field of lipase immobilization via physical adsorption can continue to evolve. This approach will ensure that new patents contribute to scientific knowledge and translate into tangible technological advancements, driving sustainable biocatalytic processes and contributing to a greener industrial landscape.

#### 4.1.4 Future Perspectives and Gaps in Biocatalysts

As biocatalysts continue to gain importance in various industrial sectors, including pharmaceuticals, food processing, and biofuels, future perspectives and identifying critical areas for advancement should be explored.^106,127,128^ A fundamental aspect is to improve biocatalysts’ efficiency, versatility, and sustainability to meet the growing demands of industrial processes.^103,129^ Research into novel enzyme engineering techniques, such as directed evolution and rational design, holds great promise for optimizing biocatalyst performance.^130,131^ In addition, the exploration of new sources of enzymes from extremophiles or genetically engineered organisms could expand the range of available biocatalysts.^132^ Despite significant progress, several challenges remain, including more cost-effective production methods, improved stability under harsh conditions, and greater substrate specificity.^133,134^ Addressing these challenges will require interdisciplinary collaboration among biologists, chemists, and engineers and continued investment in research and development.^135−137^ By overcoming these obstacles, biocatalysts can establish themselves as essential tools for sustainable and efficient industrial processes.^138,139^

Thus, one of the future perspectives for biocatalysts is related to the immobilization technique, lipase type, and the method chosen.^140^ A key point in this scenario is the support materials for enzyme immobilization, as their characteristics can improve the association with the technique and favor the catalysis process.^37^ In addition, this can contribute to cost reduction on an industrial scale.^141,142^ In this context, magnetic nanoparticles, graphene oxide and agroindustrial residues represent promising alternatives for future research.^143,144^

According to Heidarizadeh et al. (2021), lipases immobilized on nanostructured supports showed increased surface area and catalyst reusability, yielding impressive results. By incorporating magnetism into the materials, a promising avenue for future studies is using graphene oxide. Graphene oxide has a large surface area, abundant oxygen-containing functional groups (e.g., epoxy, hydroxyl, and carboxyl groups), and the ability to disperse in water, making it promising for immobilizing commercially relevant enzymes.^145^

A second alternative is agroindustrial waste, which can make processes more cost-effective and help valorize these materials, as they often lack proper disposal methods. According to Keijer et al. (2019), these materials are rich in bioactive components that can contribute to enzyme immobilization.^146−148^ Therefore, studies in this area would expand the database and enable process optimization, making large-scale industrial applications feasible. This approach is a sustainable alternative, as it could significantly reduce discarded waste by converting it into a high-value feedstock.

Focusing specifically on the immobilization of lipase by physical adsorption, several critical areas warrant deeper scientific discussion. Current methodologies primarily emphasize selecting appropriate support materials and optimizing adsorption conditions to enhance enzyme stability and activity. However, significant challenges remain, particularly regarding enzyme desorption under fluctuating operational conditions such as changes in pH, temperature, and agitation.

Recent advancements have shown promising results in hybrid approaches that combine physical adsorption with covalent binding techniques to mitigate enzyme desorption issues. While these hybrid strategies retain the advantages of mild immobilization conditions, they also provide a stronger attachment to the support, thus enhancing the operational stability of the biocatalysts. However, scaling these techniques for industrial applications remains challenging due to the complexity and cost of preparing advanced support materials.

The integration of magnetic nanoparticles and graphene oxide in support materials has been particularly noteworthy. These materials offer enhanced surface areas and unique interaction capabilities, significantly improving enzyme loading and stability. Yet, these materials’ high cost and technical complexity pose significant barriers to their widespread adoption in industrial settings. Addressing these issues requires a concerted effort to develop more cost-effective synthesis and scalable production techniques.

Furthermore, using agroindustrial residues as support materials represents a highly promising and sustainable alternative. These materials offer a low-cost solution and contribute to waste valorization, thus addressing economic and environmental concerns. Future research should focus on optimizing the properties of these residues to enhance their performance as immobilization supports, potentially through chemical modifications or the incorporation of functional groups that improve enzyme binding.

Interdisciplinary collaborations integrating insights from materials science, nanotechnology, and molecular biology will be essential to drive innovation in this field. By leveraging advanced characterization techniques and computational modeling, researchers can gain a deeper understanding of the molecular interactions between lipases and support materials, paving the way for more effective immobilization strategies. Addressing these challenges and leveraging technological advancements will be crucial for optimizing the immobilization of lipases by physical adsorption for broader industrial applications, ultimately contributing to more sustainable and efficient biocatalytic processes.

## 5 Strengths and Limitations

This study presents a number of notable strengths that significantly contribute to understanding lipase immobilization by physical adsorption. First, systematic analyses of global trends over the past 13 years were conducted using advanced bibliometric methods, providing valuable insights for researchers and highlighting important areas for future investigation. The use of widely recognized bibliometric software tools, such as VOSviewer and CiteSpace, facilitated robust analysis of WoS data and provided a deeper understanding of the dynamics of the field.

In addition, the study stands out for its rigorous application of analyses, providing a detailed view of trends in lipase immobilization related to physical adsorption. The bibliometric tools were instrumental in clearly visualizing the relationships between articles and the most relevant keywords. This enriched the understanding of the current landscape and helped identify knowledge gaps and promising directions for future research. In addition, the approach allowed the quantification and identification of institutions, authors, and countries involved, facilitating the understanding of collaborative networks and mapping emerging areas of interest. These elements were essential for assessing the impact of recent technologies and highlighting opportunities for developing new investigations and advancements in lipase immobilization via physical adsorption.

While several advantages are highlighted in this context, it is essential to recognize some significant limitations. First, there has been a decrease in the number of publications on the subject, probably because of the rapid evolution of technologies and the diversity of methods available. This has led to a wide range of study options. This decrease may also correlate with the decline in the number of patents, as shown in Figure 11, where 2023 is the lowest number in the last 13 years. These considerations highlight the complexity and diversity of approaches in the field, which require careful analysis to determine the most appropriate strategy for each research or application context. In addition, several immobilization methods are based on physical and chemical interactions between biomolecules and supports. These include physical adsorption (involving hydrophobic and van der Waals interactions), chemical adsorption (involving covalent and ionic bonds), immobilization by confinement in a matrix or microcapsule, and cross-linking.^80,149−151^

Focusing on the immobilization of lipase by physical adsorption, this study effectively elucidates the importance of supporting material selection and optimizing adsorption conditions. Despite the advantages of physical adsorption, such as simplicity and mild immobilization conditions, challenges remain in achieving high stability and preventing enzyme desorption. The study’s use of advanced bibliometric tools highlights significant trends and collaborative networks, crucial for driving innovation in this field.

However, the variability of publication and patent trends suggests that the field is still evolving, with new methodologies and materials continually being explored. This variability underscores the need for ongoing research to refine immobilization techniques and address current limitations. Future studies should focus on integrating interdisciplinary approaches, combining insights from materials science, nanotechnology, and computational modeling to enhance the efficiency and robustness of lipase immobilization.

Moreover, addressing the challenges of enzyme desorption and operational stability will be crucial for the broader industrial application of immobilized lipases. Innovative support materials such as magnetic nanoparticles and graphene oxide offer promising solutions but require further development to reduce costs and improve scalability. The field can achieve significant advancements by fostering collaborative research and leveraging advanced technologies, ultimately contributing to more sustainable and efficient biocatalytic processes.

## 6 Mechanism for Immobilization

The mechanism of enzyme immobilization is fundamental in various fields, such as medicine, engineering, and biotechnology. Enzyme immobilization involves the attachment or confinement of enzymes to a support matrix, which enhances their stability and reusability in biocatalytic processes such as biofuel and biolubricant production.^152^

The choice of support matrix plays a fundamental role in enzyme immobilization. Support materials can range from natural polymers such as agarose and chitosan to synthetic materials such as polyacrylamide and polystyrene to metallic materials coated with active enzyme surface materials.^153,154^ Examples include magnetic materials coated with active agents such as polyethylenimine, epoxy groups, and glutaraldehyde.^155^ Each matrix offers properties influencing factors such as enzyme loading capacity, stability, and compatibility with the reaction environment.^156^

The main methods for immobilizing enzymes on supports include physical adsorption, covalent binding, cross-linking, and encapsulation. Physical adsorption involves the noncovalent attachment of enzymes to the support surface, offering simplicity but often limited stability.^157^ Covalent binding consists of forming strong chemical bonds between the enzyme and the support, providing excellent stability but requiring careful optimization to prevent enzyme inactivation.^158^ Cross-linking physically traps enzymes within the support matrix, providing protection but potentially limiting mass transfer.^159^ Encapsulation, a cross-linking variation, involves encapsulating enzymes within semipermeable membranes, providing increased stability and protection from harsh reaction conditions.^160^

Factors that affect immobilization efficiency include parameters such as pH, temperature, enzyme concentration, and choice of immobilization method.^161−163^ These factors must be carefully optimized to maximize enzyme loading and activity while maintaining stability and specificity.^164^ Once immobilized, enzymes exhibit altered kinetic properties compared to their free counterparts.^165^ Factors such as diffusion limitations, substrate accessibility, and conformational changes can affect catalytic efficiency, requiring thorough characterization and optimization of immobilized enzyme systems.^166^

Understanding the mechanisms of enzyme immobilization is critical to realizing the full potential of biocatalysis in various industrial applications. Researchers can develop robust and efficient enzyme immobilization strategies tailored to specific biocatalytic processes by carefully selecting support materials, immobilization methods, and optimization parameters. This paves the way for sustainable and environmentally friendly technological advances. Table 6 briefly summarizes the main immobilization mechanisms, their advantages and disadvantages, and the main supports used for each mechanism.

**Table 6:** Main Mechanisms of Enzyme Immobilization: Advantages, Disadvantages, and Main Supports Used for Each Mechanism

immobilization mechanisms	advantages	disadvantages	main supports	references
Physical adsorption	Simple, easy to perform.	Limited stability, potential enzyme leaching.	Agarose, silica gel, activated carbon.	(157,167)
Covalent binding	Strong, stable attachment.	Requires optimization to prevent enzyme inactivation.	Glyoxyl-agarose, epoxy-activated supports, aldehyde-functionalized supports.	(158,168)
Cross-linking	Protection against harsh conditions.	Mass transfer limitations.	Alginate, polyvinyl alcohol, chitosan.	(159,169)
Encapsulation	Enhanced stability, protection.	Potential diffusion limitations.	Polymeric membranes, silica nanoparticles.	(160,170)

In physical adsorption, a critical examination of current methodologies reveals strengths and challenges. Physical adsorption is favored for its simplicity and the mild conditions it operates, making it particularly suitable for sensitive enzymes like lipases. The method relies on noncovalent interactions such as hydrophobic forces, van der Waals forces, and hydrogen bonds to attach the enzyme to the support. While these interactions are generally weak, they can be collectively sufficient to stabilize the enzyme in an immobilized state under optimal conditions.

However, one of the primary challenges of physical adsorption is the potential for enzyme desorption, especially under varying operational conditions such as changes in pH, temperature, or mechanical agitation. This can lead to a loss of enzymatic activity and reduced reusability of the biocatalyst. To address this, researchers are exploring hybrid approaches that combine physical adsorption with other immobilization techniques, such as covalent binding, to enhance the strength of enzyme attachment.

Recent advancements in support materials have also shown promise in overcoming some limitations of physical adsorption. For instance, magnetic nanoparticles and graphene oxide provide large surface areas and unique interaction properties that can enhance enzyme loading and stability. Additionally, functionalizing these materials with specific groups can improve the binding affinity for enzymes, reducing the likelihood of desorption. Despite these advancements, the cost and complexity of preparing these advanced materials remain significant challenges that must be addressed for large-scale applications.

Furthermore, integrating computational tools and AI in designing and optimizing immobilization processes offers new avenues for improving efficiency and effectiveness. By simulating enzyme-support interactions at the molecular level, researchers can predict the optimal conditions for immobilization and identify the most suitable support materials and methods.

Overall, while physical adsorption remains a viable and widely used method for enzyme immobilization, ongoing research and technological advancements are essential to address its limitations and enhance its applicability. By continuing to innovate and optimize immobilization strategies, the field can achieve significant progress in developing robust and efficient biocatalysts for a wide range of industrial applications, contributing to more sustainable and environmentally friendly processes.

### 6.1 Support Specificity

A fundamental aspect of enzyme immobilization is the choice of immobilization support, which plays a central role in the efficiency and stability of biocatalysis. Immobilization supports can be divided into two categories: natural and synthetic. Natural supports include alginate, cellulose, and chitosan, which offer advantages such as biocompatibility, low cost, and easy availability.^171^ However, synthetic supports, such as acrylic polymers and modified silica, provide greater control over the physicochemical properties of the support, allowing precise tailoring to the specific needs of the immobilized enzyme.^172,173^

The choice of the ideal support depends on several factors, including the nature of the enzyme, the reaction conditions, and the desired properties of the immobilized biocatalyst. For example, enzymes with specific functional groups may selectively interact with certain chemical groups on the support surface, promoting immobilization by covalent bonding or adsorption.^174^ A suitable surface should provide binding sites for enzyme attachment and facilitate stable interactions that prevent enzyme leaching during the catalytic process. In addition, the morphology and porous structure of the support plays a crucial role in the immobilization efficiency. Supports for adequate porosity facilitate substrate and product diffusion, while materials with high surface area provide more excellent enzyme–substrate contact, thereby increasing biocatalytic efficiency.^174,175^

Advanced support design strategies, such as chemical modification of the support surface and incorporation of specific functional groups, allow fine-tuning of support properties to optimize enzyme immobilization. In addition, innovative approaches (e.g., hybrid and nanostructured supports) offer new opportunities to develop highly efficient, stable immobilization systems.

In the context of physical adsorption, the specificity of the support material is paramount to the success of the immobilization process. The interaction between the enzyme and the support is primarily governed by noncovalent forces such as hydrophobic interactions, hydrogen bonds, and van der Waals forces. Therefore, the support choice must ensure that these interactions are strong enough to maintain enzyme stability and activity during the catalytic process.

Natural supports, while advantageous for their biocompatibility and cost-effectiveness, often lack the mechanical strength and durability required for industrial applications. On the other hand, synthetic supports offer customizable properties, such as controlled pore sizes, surface areas, and functional groups, which can be tailored to enhance enzyme-support interactions. For instance, modified silica and acrylic polymers can be engineered to present hydrophobic or hydrophilic surfaces that match the enzyme’s requirements, thereby enhancing immobilization efficiency and stability.

The morphology of the support also plays a crucial role in determining the immobilization outcome. Supports with high surface areas and appropriate pore sizes facilitate better enzyme loading and accessibility, leading to higher catalytic efficiencies. Moreover, the mechanical properties of the support must be considered to ensure that they can withstand the operational conditions without degrading or losing structural integrity.

Innovative support materials, such as magnetic nanoparticles and graphene oxide, have shown significant promise in improving the efficiency of enzyme immobilization. These materials offer unique properties, such as high surface areas, tunable surface chemistry, and ease of recovery through magnetic separation. However, their high cost and complex preparation processes remain challenges that must be addressed for widespread industrial application.

Future research should focus on developing cost-effective and scalable methods for synthesizing advanced support materials. Additionally, integrating computational modeling and AI-driven optimization can help predict the best combinations of support properties and immobilization conditions, leading to more efficient and robust immobilization strategies. By addressing these challenges, the field can achieve significant advancements in enzyme immobilization, ultimately contributing to more sustainable and efficient biocatalytic processes across various industries.

## 7 Advantages

Enzymes are widely used in several large and medium-sized industries, including the production of biofuels, detergents, animal feeds, and food-based products (e.g., dairy products, baked goods, and fruit juices) as well as other applications such as paper, leather, and textile processing.^176−178^ Lipases are widely distributed in plants, animals, insects, and microorganisms such as bacteria and fungi. These enzymes catalyze the hydrolysis of triglycerides, esterification, transesterification, and reactions on unnatural substrates.^178−180^ Lipase (EC 3.1.1.3) is used commercially in food processing, particularly in producing structured lipids as dietary ingredients derived from vegetable and animal fats and oils.^176−178,181^

Immobilization positively affects lipase stability at high temperatures, with immobilized lipase exhibiting more significant activity than free lipase.^180,182−184^ These enzymes can be immobilized in various ways using the methods mentioned above. This review presents the most recent studies on enzyme adsorption on hydrophobic solid support with low acquisition and operating costs.^184−186^ Lipase immobilization via physical adsorption occurs primarily through physical interactions involving noncovalent forces such as van der Waals forces, London dispersion, and hydrophobic interactions between the enzymes and the support surface material.^187−192^

The main advantages include simplicity, low cost, and minimal effect on the activity of the enzymes as they do not undergo significant conformational or chemical changes.^190−195^ In particular, storage and operational stability of the immobilized lipase are critical factors for industrial applications.^193,195−198^ While high temperature minimizes lipase activity, it increases thermal stability, providing more excellent operational stability.^189−195^

Immobilization by physical adsorption improves the properties of the enzyme, increasing its rigidity and heat tolerance and providing a broader range of activity. This is because of the low conformational flexibility of the enzyme, which is indicated by an increase in the optimal temperature and stability against inactivation.^183−186,188^ The improved thermal behavior of immobilized enzymes is the basis for many industrial processes requiring high temperatures.^178,182^ However, the strength of the immobilized enzyme may be compromised because of the limited, weak nature of these interactions.^176,177^

In this sense, immobilized enzymes are more versatile because they can function in more aggressive environments that might otherwise affect the enzyme’s activity. With physical adsorption, immobilization costs are much lower than other classical enzyme immobilization methods.^176−178,199^ Enzymes can be adsorbed onto hydrophobic supports and undergo interfacial activation during adsorption.^178,181,199^

Porous supports offer many advantages over nonporous supports.^177,199^ Numerous studies have reported that porous supports have a better surface area and can transport more enzymes with higher immobilization rates, thus providing more regions for direct enzyme immobilization without the use of reagents or ligands.^179,182,184,185,199^ Consequently, the adsorption of enzymes onto porous supports with hydrophobic properties improves the reusability, pH stability, thermostability, and operational and storage stability of lipase for long-term, large-scale applications.^184−187^

Because of their easy recovery and reuse, which reduces the cost of industrial applications, enzymes immobilized by physical adsorption — prepared by binding free enzymes to solid supports — have received increasing attention from manufacturers and researchers for their innovative and promising potential.^186,188−190^ Immobilization by physical interaction can provide excellent stability and reusability, allowing easier enzyme recovery and higher enzymatic activity.^193−196^ Studies report the retention of more than 50% of the enzymatic activity of the biocatalyst formed by physical interaction immobilization after seven reaction cycles. This sustained activity is increasing industry interest in large-scale applications of these physical biocatalysts.^190−192,194^

In summary, immobilization via physical adsorption can improve enzymes’ thermal and operational stability, providing a protective environment that protects them from degradation and denaturation.^192−196^ Furthermore, one of the main advantages of immobilized enzymes is their reusability, which can significantly reduce the cost of enzyme-catalyzed reactions in industrial applications.^182,185−188,190,192^ Immobilized enzymes can be easily separated from the reaction mixture and reused multiple times without significant activity loss.^176−178^

A critical analysis of current methodologies for immobilizing lipases via physical adsorption reveals significant advancements and persistent challenges. The simplicity and cost-effectiveness of this technique make it highly attractive for industrial applications. However, the noncovalent nature of the interactions involved in physical adsorption, while beneficial for maintaining enzyme activity, often results in weaker binding forces compared to covalent methods. This can lead to enzyme leaching and reduced long-term stability under varying operational conditions.

Recent advancements have focused on optimizing support materials to enhance the strength of enzyme-support interactions. Advanced materials such as magnetic nanoparticles and graphene oxide have shown promise in providing robust and stable immobilization platforms. These materials offer high surface areas and functionalizable surfaces, which can be tailored to improve enzyme loading and stability. Despite these advantages, these materials’ high cost and complex synthesis processes remain barriers to their widespread industrial adoption.

Innovative strategies, such as hybrid immobilization techniques that combine physical adsorption with covalent binding, have been developed to address the limitations of each method. These hybrid approaches leverage the strengths of both techniques, providing strong enzyme attachment and preserving enzyme activity. Additionally, incorporating computational modeling and AI-driven optimization can further refine these methods, predicting optimal conditions and supporting material properties for specific applications.

Future research should aim to develop scalable and economically viable methods for producing advanced support materials and hybrid immobilization techniques. Interdisciplinary collaborations integrating insights from materials science, nanotechnology, and computational modeling will be crucial in driving innovation in this field. By addressing these challenges, the immobilization of lipases via physical adsorption can be further optimized, contributing to more sustainable and efficient biocatalytic processes across various industries.

## 8 Challenges

Enzyme immobilization via physical adsorption is considered highly relevant and impactful in the biotechnology industry because of its simplicity and low cost.^200,201^ However, there are obstacles to overcome, such as a decrease in enzymatic activity caused by factors such as loss of contact with the support or changes in the enzyme^202−204^ microenvironment. These changes in the microenvironment are often because of enzyme rearrangements that alter the availability of active sites for catalyzing reactions.^205,206^

In addition, the biological catalyst may be released during the reaction due to the noncovalent bond between the enzyme and the carrier.^207,208^ In pharmaceutical and food applications, ensuring product quality and safety is imperative. Noncompliance can lead to regulatory issues and even product withdrawal from the market in.^209−211^ Competition between enzymes for the desired substrate can affect reaction yield, potentially leading to the formation of unwanted byproducts, changes in the properties of the final product, and, consequently, an increase in production costs.^212,213^

The pH and temperature are critical factors in immobilization and significantly affect stability.^214^ pH of the reaction must be appropriately adjusted based on the characteristics of the enzyme to optimize adsorption and to affect the surface charge of the enzyme-support complex, which influences their interaction.^215−217^ In addition, significant temperature changes can alter the conformation and activity of the enzyme, potentially causing denaturation and disruption of noncovalent bonds within the enzyme-support complex.^218−220^

Another challenge in immobilizing enzymes by physical adsorption is limited diffusion. Noncovalent interactions (e.g., van der Waals and hydrogen bonds) can affect mobility by restricting the porous structure of the support.^221−223^ As a result, barriers to the diffusion of substrates and products are created, resulting in lower reaction rates because of reduced catalytic efficiency.^224,225^ In support matrices with insufficient porosity, substrate access to the enzyme catalytic site is limited.^226,227^ In systems with high enzyme concentrations, this can lead to competition for adsorption sites and the formation of enzyme clusters.^228^

Focusing specifically on the challenges associated with lipase immobilization via physical adsorption, several critical issues warrant deeper examination. This method’s simplicity and low cost are balanced by the inherent weaknesses of noncovalent interactions, which can lead to enzyme desorption and loss of activity over time. This issue is particularly significant in industrial applications where long-term stability and reusability are crucial.

One major challenge is maintaining the enzyme’s active conformation upon immobilization. Physical adsorption relies on weak forces easily disrupted by environmental changes such as pH shifts and temperature fluctuations. This can result in the enzyme adopting an inactive conformation or denaturing, drastically reducing catalytic efficiency. Strategies to mitigate this include optimizing the immobilization conditions to favor strong enzyme-support interactions and selecting supports with surface chemistries that stabilize the active enzyme conformation.

Another significant challenge is the diffusion limitation imposed by the support material. The porous structure of the support is critical for allowing substrates to reach the immobilized enzyme’s active sites. However, if the pores are too small or the distribution is not optimal, substrate diffusion can be severely restricted, leading to lower reaction rates. Advanced materials such as mesoporous silica and hierarchical structures can offer improved diffusion properties, but their synthesis and functionalization must be carefully controlled to ensure consistency and performance.

The potential release of the enzyme from the support during the reaction is another hurdle. This can occur due to the weak nature of noncovalent bonds, especially under high shear or agitation conditions commonly found in industrial processes. Hybrid immobilization techniques that combine physical adsorption with more muscular covalent attachments or encapsulation can provide a more robust solution, enhancing the immobilized enzyme’s stability and reusability.

Furthermore, other biomolecules or contaminants in the reaction mixture can influence the interaction between the enzyme and the support. These can compete for adsorption sites or interact with the enzyme in ways that reduce its activity. Purifying the enzyme prior to immobilization and using selective support materials that preferentially bind the enzyme can help address this issue.

Future research should focus on developing more sophisticated support materials and immobilization techniques that address these challenges. Innovations in material science will be critical, such as creating responsive supports that can adapt to environmental changes and integrating computational tools to predict optimal immobilization conditions. By overcoming these challenges, the immobilization of lipases via physical adsorption can be further optimized, enhancing their utility in various industrial applications.

## 9 Solutions

### 9.1 Implementation of Solutions to Overcome Challenges

#### 9.1.1 Improved Support

By continuing the study of enzyme immobilization, we will present solutions to the potential problems encountered in this process.^229^ The first approach is to improve the support, which is fundamental in enzyme immobilization as it directly affects the stability, activity, and reuse of these enzymes.^230,231^ Several methods exist to improve the support used and optimize the performance of immobilized enzymes.^232,233^

One of the most common methods is chemical modification of the support, which involves adding functional groups to the support to increase its affinity for the enzyme.^234^ This process is carried out by introducing amino, carboxylic or sulfonic groups that provide specific interactions between the enzyme and the support.^231,235,236^

In addition, materials engineering offers opportunities to develop more stable and resilient supports.^237^ By using techniques such as nanotechnology, it is possible to create supports with controlled porous structures and increased surface area. This provides a more favorable environment for immobilization and improves the stability of the enzyme.^238^

Another strategy is to carefully select the support to be used, as this selection plays a prominent role in the efficiency of the immobilization process.^239^ Materials such as cellulose, chitosan, silica, and synthetic polymers are commonly used due to their favorable physical and chemical properties.^240^ Therefore, considerations for support selection are based on the concept that the appropriate support depends on the characteristics of the enzyme, the process, and the operating conditions.^241^

Focusing specifically on improving supports for lipase immobilization via physical adsorption, several advanced strategies can be implemented to address the current challenges and enhance the overall efficiency of the process.

Chemical modification of support materials can significantly enhance the binding affinity and stability of the immobilized enzyme. For example, introducing functional groups such as amino, carboxylic, or sulfonic acids can create specific sites for enzyme attachment, promoting more robust and more stable interactions. This can be particularly effective for enzymes with complementary functional groups, facilitating covalent or ionic bonding. The choice of functional group should be tailored to the enzyme’s properties to maximize immobilization efficiency and activity.

The application of nanotechnology in materials engineering offers a promising avenue for developing advanced supports with superior properties. Nanostructured materials such as mesoporous silica, carbon nanotubes, and graphene oxide can provide a high surface area and controlled pore size distribution, which are critical for effective enzyme immobilization. These materials can also be engineered to have specific surface chemistries that enhance enzyme binding and stability. The increased surface area increases enzyme loading, while the controlled pore structure ensures adequate substrate diffusion, enhancing catalytic efficiency.

The careful selection of support materials is crucial for optimizing the immobilization process. Natural polymers like cellulose and chitosan are favored for their biocompatibility and ease of modification. On the other hand, synthetic polymers offer greater control over physical and chemical properties, allowing for the design of tailored supports that meet specific immobilization requirements. Silica-based materials are particularly advantageous due to their mechanical strength, thermal stability, and ease of functionalization. The choice of support material should consider factors such as enzyme stability, process conditions, and desired product properties.

To further improve support materials, advanced characterization techniques such as scanning electron microscopy (SEM), transmission electron microscopy (TEM), and atomic force microscopy (AFM) can be used to analyze the surface morphology and porosity of the supports. Additionally, computational modeling and simulation can predict the interactions between the enzyme and the support, providing insights into optimal immobilization conditions. These approaches enable the rational design of support materials that maximize enzyme loading and stability while minimizing mass transfer limitations.

Future research should focus on developing scalable and cost-effective methods for producing advanced support materials. Integrating multidisciplinary approaches, combining insights from materials science, nanotechnology, and computational modeling, will be essential for driving innovation in this field. By addressing the current challenges and leveraging advanced technologies, the immobilization of lipases via physical adsorption can be further optimized, contributing to more efficient and sustainable biocatalytic processes across various industries.

#### 9.1.2 Combined Immobilization

Besides selecting the appropriate support, other methods can improve the immobilization process or the biocatalyst produced.^242^ For example, combined immobilization is a biotechnological method in which more than one method is used to immobilize enzymes.^243^ This approach takes advantage of different immobilization techniques to improve the performance of enzymes in a given system.^244^

In the combined immobilization process, chemical and physical methods are expected to produce a single biocatalyst. For example, the combination of adsorption (a physical method) and covalent bonding (a chemical process) would proceed: the enzyme would first be adsorbed onto a solid support, followed by covalent bonds between the enzyme and the support.^245^ This approach combines the simplicity of the adsorption method with the stability provided by covalent bonds, resulting in a more robust immobilization.^246^

Besides using two immobilization methods, another strategy is to combine different supports. Each support has unique characteristics, such as high surface area or good thermal stability.^247^ Combining these supports makes it possible to create an immobilization system that maximizes enzyme activity and stability under well-defined application conditions.^248^

Besides the methods already described, combined immobilization may also involve using additional materials to enhance the system’s performance.^249^ For example, nanoparticles can be added to the solid support to increase the contact surface area available for immobilization or to provide specific binding sites for the enzyme.^250^ This approach allows the properties of the immobilization system to be tailored to the particular needs of a biotechnological application.^251^Table 7 presents a selection of articles that use the enzymatic coimmobilization method to achieve better yield results.

**Table 7:** Ranking of the 10 Most Referenced Articles That Employ the Technique of Enzyme Coimmobilization

rank	titles	authors	journals	year of publication	no. of citations	references
1	Self-Assembling Protein Scaffold System for Easy in Vitro Coimmobilization of Biocatalytic Cascade Enzymes.	Zhang, G., Quin, M.B., Schmidt-Dannert, C.	ACS Catalysis	2018	114	(244)
2	Development of simple protocols to solve the problems of enzyme coimmobilization. Application to coimmobilize a lipase and a β-galactosidase.	Peirce, S., Virgen-Ortíz, J.J., Tacias-Pascacio, V.G., Marzocchella, A., Fernandez-Lafuente, R.	RSC Advances	2016	94	(243)
3	Taguchi design-assisted coimmobilization of lipase A and B from *Candida antarctica* onto chitosan: Characterization, kinetic resolution application, and docking studies.	da S. Moreira, K., Barros de Oliveira, A.L., Simao Neto, F., Marques da Fonseca, A., dos Santos, J.C.S.	Chemical Engineering Research and Design	2022	64	(50)
4	Coimmobilization of a redox enzyme and a cofactor regeneration system.	Betancor, L., Berne, C., Luckarift, H.R., Spain, J.C.	Chemical Communications	2006	72	(245)
5	High Activity and Convenient Ratio Control: DNA-Directed Coimmobilization of Multiple Enzymes on Multifunctionalized Magnetic Nanoparticles.	Yang, Y., Zhang, R., Zhou, B., Su, P., Yang, Y.	ACS Applied Materials and Interfaces	2017	53	(246)
6	Coimmobilization of enzymes in bilayers using pei as a glue to reuse the most stable enzyme: Preventing pei release during inactivated enzyme desorption.	Zaak, H., Kornecki, J.F., Siar, E.-H., Sassi, M., Fernandez-Lafuente, R.	Process Biochemistry	2017	45	(247)
7	Advantages of Supports Activated with Divinyl Sulfone in Enzyme Coimmobilization: Possibility of Multipoint Covalent Immobilization of the Most Stable Enzyme and Immobilization via Ion Exchange of the Least Stable Enzyme.	Morellon-Sterling, R., Carballares, D., Arana-Peña, S., Braham, S.A., Fernandez-Lafuente, R.	ACS Sustainable Chemistry and Engineering	2021	38	(248)
8	Coimmobilization of different lipases: Simple layer by layer enzyme spatial ordering.	Arana-Peña, S., Rios, N.S., Mendez-Sanchez, C., Gonçalves, L.R.B., Fernandez-Lafuente, R.	International Journal of Biological Macromolecules	2020	38	(249)
9	The combination of covalent and ionic exchange immobilizations enables the coimmobilization on vinyl sulfone activated supports and the reuse of the most stable immobilized enzyme.	Arana-Peña, S., Carballares, D., Morellon-Sterling, R., Rocha-Martin, J., Fernandez-Lafuente, R.	International Journal of Biological Macromolecules	2022	28	(250)
10	Multifunctional magnetic particles for effective suppression of nonspecific adsorption and coimmobilization of multiple enzymes by DNA directed immobilization.	Song, J., Shen, H., Yang, Y.,···Su, P., Yang, Y.	Journal of Materials Chemistry B	2018	24	(251)

Focusing specifically on the immobilization of lipase by physical adsorption, the combined immobilization approach offers a promising strategy to enhance enzyme stability, activity, and reusability. Physical adsorption alone often suffers from weak interactions between the enzyme and the support, leading to potential enzyme leaching and reduced stability. These limitations can be mitigated by integrating it with other methods like covalent bonding or hybrid supports.

Current methodologies for combined immobilization involve a multistep process where physical adsorption is followed by chemical stabilization. For instance, initially adsorbing lipase onto hydrophobic support can orient the enzyme for optimal activity, while subsequent covalent bonding secures it, preventing desorption under operational conditions. This hybrid approach leverages the ease and mild adsorption conditions with the covalent attachment robustness.

However, challenges remain in achieving uniform enzyme distribution and maintaining enzyme activity postimmobilization. Nonuniform distribution can lead to areas of high enzyme density, causing diffusion limitations and reduced overall activity. Additionally, the immobilization process can sometimes lead to partial denaturation of the enzyme, affecting its catalytic efficiency.

Recent advances in support materials have shown promise in addressing these challenges. Nanoparticles, for instance, offer a high surface area and unique physicochemical properties that enhance enzyme-support interactions. Magnetic nanoparticles, in particular, facilitate easy recovery and reuse of immobilized enzymes, making them attractive for industrial applications. Graphene oxide is another material that has garnered attention due to its large surface area and ability to form stable enzyme interactions.

Combining these advanced materials with traditional supports can create hybrid systems that maximize both benefits. For example, combining porous silica with magnetic nanoparticles can provide high enzyme loading and ease of separation. Such hybrid supports can also be engineered to possess specific functional groups that enhance enzyme binding and stability.

Future research should optimize the conditions for combined immobilization to ensure maximal enzyme activity and stability. This includes fine-tuning the adsorption conditions (e.g., pH, ionic strength) and the subsequent chemical stabilization steps (e.g., choice of cross-linkers, reaction time). Additionally, exploring the synergistic effects of combining different support materials can lead to the development more efficient immobilization systems.

Integrating computational modeling and machine learning can also be crucial in predicting the optimal conditions for enzyme immobilization. By analyzing large data sets from experimental studies, these tools can help identify patterns and conditions that lead to improved enzyme performance. Addressing these challenges and leveraging recent advances in materials science and biotechnology can significantly improve the immobilization of lipases via combined methods. This approach enhances the stability and activity of immobilized enzymes and expands their applicability in various industrial processes, contributing to more sustainable and efficient biocatalytic solutions.

#### 9.1.3 Optimization of Immobilization Conditions

To further solve the problems of enzyme immobilization, we now present the optimization of immobilization conditions.^252,253^ This biotechnological process is essential to maximize the efficiency and stability of enzymes in immobilized systems.^254,255^ The goal is to adjust various parameters to achieve optimal performance of both immobilization and catalytic activity of the enzyme.^251,256^ These parameter adjustments include the appropriate support choice, immobilization method, reaction conditions, and binding agents.^257,258^

As discussed in this paper, the support is a fundamental part of the immobilization.^259,260^ The support should be selected based on its ability to provide a high surface area, good mechanical and chemical stability, and easy accessibility of substrates to the enzyme.^261,262^ Furthermore, the immobilization method must be carefully defined and applied to ensure uniform enzyme distribution on the support and to avoid denaturation or inactivation during the process.^263,264^ Reaction conditions (e.g., pH, temperature, and substrate concentration) must also be optimized to maximize the catalytic activity of the immobilized enzyme.^265,266^

Furthermore, binders are used to provide more excellent stabilization of the enzyme on the support to prevent damage from leaching or unwanted release of the enzyme from the support.^267^ Optimization of these parameters improves the performance of the immobilized enzyme in terms of activity and stability.^268^

A critical aspect of optimizing immobilization conditions for lipase enzymes via physical adsorption involves a detailed understanding of how different parameters affect the stability and activity of the immobilized enzyme. Here, we delve deeper into the specific strategies and scientific principles that guide this optimization process.

The choice of support material is crucial for successful enzyme immobilization. Supports must possess high surface areas to accommodate many enzyme molecules. For instance, mesoporous materials like silica, alumina, and zeolites are often preferred due to their extensive surface areas and tunable pore sizes, facilitating high enzyme loading and substrate accessibility. Moreover, the support’s mechanical strength and chemical stability are critical to maintaining enzyme activity under industrial processing conditions.

The conditions under which immobilization occurs can significantly influence the enzyme’s performance. Parameters such as the pH and ionic strength of the immobilization medium can affect the enzyme’s conformation and the nature of enzyme-support interactions. For example, immobilizing lipases at a pH near their isoelectric point can enhance adsorption efficiency due to minimal electrostatic repulsion between the enzyme and the support.

Temperature plays a dual role in enzyme immobilization. While elevated temperatures can increase the rate of immobilization, they can also lead to enzyme denaturation. Therefore, immobilization should be conducted at temperatures that preserve the enzyme’s native conformation. Postimmobilization, the operational stability of the enzyme at different temperatures must be assessed to ensure that the immobilized enzyme retains its activity under the desired reaction conditions.

Binding agents or linkers can enhance the stability of the immobilized enzyme. Agents such as glutaraldehyde or carbodiimides can form covalent bonds between the enzyme and the support, reducing the likelihood of enzyme leaching. The choice and concentration of these agents must be optimized to balance strong enzyme-support attachment and retention of enzyme activity.

The substrate concentration during the immobilization process should be optimized to ensure maximal enzyme activity. Additionally, the diffusion of substrates and products in and out of the porous support must be facilitated. Supports with appropriate pore sizes and structures can minimize diffusion limitations, thereby enhancing the overall catalytic efficiency of the immobilized enzyme.

Optimizing immobilization conditions requires a comprehensive understanding of the interactions between the enzyme, support, and the surrounding environment. Advanced analytical techniques, such as surface plasmon resonance (SPR) and quartz crystal microbalance (QCM), can provide real-time insights into these interactions, aiding in fine-tuning immobilization parameters.

Future research should focus on developing high-throughput screening methods for rapidly optimizing immobilization conditions. Additionally, integrating computational modeling and machine learning algorithms can predict optimal conditions based on the physicochemical properties and support of the enzyme, thus accelerating the optimization process. By addressing these challenges and leveraging advanced technologies, optimizing immobilization conditions can significantly enhance immobilized lipases’ stability, activity, and reusability, paving the way for more efficient and sustainable industrial biocatalytic processes.

#### 9.1.4 Enzyme Recycling

Improving the stability of a biocatalyst will facilitate its recycling process. Enzyme recycling involves using an enzyme in a chemical reaction and then recovering and reusing it multiple times, reducing costs and making processes more sustainable.^257^ Enzyme recycling enzymes are immobilized in a solid matrix, such as silica gel,^269^ nanoparticles,^270^ or cellulose.^271^ This immobilization facilitates their separation from the reaction medium and allows them to be reused.^272^

This reuse offers several advantages over the use of soluble enzymes.^273^ Because immobilized enzymes are more stable and resistant to changes in pH and temperature, their useful life is extended. Therefore, immobilized enzymes may be reused for multiple reactions.^274^

However, it is essential to note that enzyme recycling is not always efficient.^275^ Over time, the catalytic activity of enzymes can decrease because of factors such as protein denaturation or loss of catalytic activity during reaction cycles.^276^ Therefore, it is necessary to optimize reaction conditions and immobilization methods to ensure maximum efficiency in enzyme recycling. In addition, enzyme reuse represents another significant advance in studies in this research field.^277^

Focusing specifically on the immobilization of lipases via physical adsorption, enzyme recycling presents opportunities and challenges that must be critically examined. The physical adsorption method relies on weak, noncovalent interactions such as van der Waals forces, hydrogen bonds, and hydrophobic interactions. While beneficial for maintaining enzyme activity due to minimal conformational changes, these interactions can also result in enzyme leaching and reduced long-term stability.

One of the main challenges in enzyme recycling is maintaining the enzyme’s stability and activity over multiple cycles. The weak interactions facilitating the initial immobilization can become a liability during extended use, as environmental fluctuations such as pH changes and mechanical agitation can disrupt the enzyme-support bond. To address this, optimizing the immobilization conditions is crucial. This includes selecting supports with surface chemistries that enhance enzyme affinity and stability and fine-tuning reaction conditions to minimize stress on the enzyme.

Advanced materials such as magnetic nanoparticles and mesoporous silica have shown promise in enhancing enzyme immobilization. Magnetic nanoparticles facilitate easy recovery of immobilized enzymes through magnetic separation, reducing mechanical stress and improving reusability. Mesoporous silica provides a high surface area and tunable pore sizes, improving enzyme loading and substrate accessibility. However, the high cost and complexity of synthesizing these materials remain barriers to widespread adoption.

Combining physical adsorption with other immobilization techniques, such as covalent bonding, can enhance the stability and reusability of immobilized enzymes. For instance, enzymes can first be adsorbed onto a support and then further stabilized through covalent bonds. This hybrid approach leverages the simplicity of physical adsorption and the robustness of covalent immobilization, resulting in a more stable biocatalyst that retains high activity over multiple cycles.

Optimizing reaction conditions, such as pH, temperature, and substrate concentration, is essential for maximizing the efficiency of enzyme recycling. Each enzyme has specific conditions under which it exhibits peak activity, and maintaining these conditions throughout the recycling process is crucial. Optimizing the immobilization conditions to ensure strong and stable enzyme-support interactions can help maintain enzyme activity over multiple cycles.

Future research should focus on developing cost-effective and scalable methods for producing advanced support materials and hybrid immobilization techniques. Integrating computational modeling and AI-driven optimization can help predict optimal conditions and support material properties for specific applications, further enhancing the efficiency of enzyme recycling. Advances in materials science, such as the development of responsive and adaptive supports, can also contribute to more effective enzyme recycling strategies.

By addressing these challenges and leveraging advanced technologies, the immobilization of lipases via physical adsorption can be further optimized, enhancing their utility in various industrial applications. This approach improves the sustainability of biocatalytic processes and reduces costs and environmental impact, making enzyme recycling a valuable strategy for the future.

#### 9.1.5 Enzyme Engineering

The growing number of studies involving enzymes has led to the development of a research field called enzyme engineering. This field of biotechnology focuses on modifying and optimizing the properties of enzymes for specific applications.^278,279^ Enzyme engineering involves manipulating the genes that encode enzymes and selecting mutations that improve their desired properties, such as stability, activity, substrate specificity, and tolerance to extreme environmental conditions.^230^

Regarding enzyme immobilization, enzyme engineering can be used to develop new, improved, and specific biocatalysts.^261^ This process involves the use of different supports and immobilization methods that can modify the structure of the enzyme to facilitate its attachment to the support material.^241^ In addition, enzyme engineering can improve the stability of biocatalysts, thereby extending their useful life during application.^273^

Enzyme engineering offers a promising avenue for enhancing the immobilization of lipases via physical adsorption, providing opportunities to tailor enzyme properties to meet specific industrial needs. By leveraging genetic manipulation and protein engineering techniques, researchers can create enzymes with enhanced characteristics that improve their performance when immobilized.

Two primary strategies in enzyme engineering are rational design and directed evolution. Rational design uses computational models and structural information to introduce specific mutations that enhance enzyme properties. This approach requires detailed knowledge of the enzyme’s structure–function relationship, enabling precise modifications to improve stability, activity, or binding affinity.

Directed evolution, on the other hand, mimics natural selection to evolve enzymes with desired traits. Researchers can identify mutants with improved properties by creating a library of enzyme variants and subjecting them to iterative rounds of mutation and selection. This method does not require prior structural knowledge and can yield enzymes with significantly enhanced performance.

Engineered enzymes can be designed to have surface-exposed residues that enhance their interaction with immobilization supports. For instance, introducing hydrophobic or charged residues at strategic locations can improve adsorption efficiency on hydrophobic or ionic supports. Additionally, enzymes can be engineered to form stronger noncovalent interactions with the support, reducing the likelihood of desorption during use.

Enzyme engineering can also enhance the stability and activity of immobilized enzymes. Mutations that increase thermal stability or denaturation resistance can extend the immobilized enzyme’s operational lifespan. Furthermore, engineering enzymes to have optimal activity under specific reaction conditions (e.g., pH, temperature) ensures that the immobilized enzyme performs efficiently in industrial processes.

Lipases, being versatile enzymes, are prime candidates for engineering. For example, *Candida rugosa* and *Candida antarctica* lipases have been engineered to improve their stability and activity when immobilized. Mutations that enhance the enzyme’s hydrophobic interactions with the support or increase its resistance to denaturation have proven effective. Additionally, engineered lipases with altered substrate specificity can be tailored for specific applications, such as biodiesel production or the synthesis of fine chemicals.

Despite the significant advancements in enzyme engineering, challenges remain. One major challenge is the potential trade-off between stability and activity, where mutations that enhance one property may negatively impact the other. Balancing these traits requires careful optimization and iterative testing.

Future research should focus on integrating enzyme engineering with advanced immobilization techniques. Combining engineered enzymes with novel supports, such as responsive or adaptive materials, can create highly efficient, stable immobilization systems. Additionally, computational tools and AI-driven optimization can help design and predict the best combinations of immobilization methods and support materials, further improving the efficiency and effectiveness of these systems.

By addressing these challenges and leveraging the potential of enzyme engineering, the immobilization of lipases via physical adsorption can be significantly improved. This approach enhances the performance and stability of immobilized enzymes and expands their applicability in various industrial processes, contributing to more sustainable and efficient biocatalytic solutions.

When specifically focusing on the immobilization of lipases by physical adsorption, it is essential to critically analyze the methodologies used, the current challenges, and the advancements made in this technique. Physical adsorption relies on weak interactions, which can lead to enzyme leaching and instability while preserving the enzyme’s active conformation. Recent advancements have sought to address these issues through the development of hybrid immobilization techniques that combine physical adsorption with stronger covalent bonding or encapsulation methods.

Moreover, the use of advanced support materials, such as magnetic nanoparticles and graphene oxide, has shown promise in enhancing the stability and activity of immobilized enzymes. These materials provide high surface areas and specific binding sites, which can improve enzyme loading and reduce the likelihood of desorption. However, the high cost and complexity of synthesizing these materials pose significant barriers to their widespread adoption.

Additionally, enzyme engineering can optimize the interaction between lipases and support materials by introducing mutations that enhance binding affinity and stability. Combining these engineered enzymes with advanced immobilization supports can result in highly efficient, stable biocatalysts over multiple reaction cycles.

Future research should continue to explore these combined approaches, focusing on developing cost-effective and scalable methods for enzyme immobilization. Integrating computational modeling and AI-driven optimization can also play a crucial role in predicting optimal immobilization conditions and designing novel support materials. By overcoming these challenges, the immobilization of lipases via physical adsorption can be further refined, offering robust and efficient biocatalysts for various industrial applications.

## 10 Conclusion

This study provides an overview of enzyme immobilization by physical adsorption, highlighting trends from 2010 to 2023. Publication growth was steady until 2022, with a slight decline in 2023, but daily updates in the Web of Science database suggest future growth. China leads in total publications and citations, followed by Brazil, showing significant interest from academia and industry. International collaboration and knowledge exchange are crucial for advancing this field.

Emerging topics like nanoparticles and magnetic materials for enzyme immobilization show promise for enhancing efficiency and stability. Review papers by Roger A. Sheldon and Mohamad Nur Royhaila offer essential insights for future research. In conclusion, enzyme immobilization by physical adsorption improves enzyme stability and reusability, making processes more economical and eco-friendly. Continued research and innovation will benefit both science and industry.

At least 10 institutions have contributed to research into the immobilization of lipases by physical adsorption, with the Federal University of Alfenas leading the way with 19 articles, followed by Tiradentes University (16) and the Consejo Superior de Investigaciones Científicas (CSIC) (15). Brazil is fourth on the list of most productive institutions. Network analyses with CiteSpace and VOSviewer highlight the collaboration between institutions, with UF Alfenas and Tiradentes University being central to the field and other emerging universities contributing to the area.

The journal Molecular Catalysis B: Enzymatic leads the publications on lipase immobilization with 17 articles, followed by the International Journal of Biological Macromolecules and Process Biochemistry with 12 publications. The International Journal of Biological Macromolecules has the highest impact factor (8.03). Publications are concentrated in three categories: Physics, Molecular Biology and Medicine. The cocitation map highlights the Journal of Molecular Catalysis B: Enzymatic as the most central, indicating that future developments will likely be published in these journals.

The immobilization of lipases via physical adsorption is valued for its simplicity, cost-effectiveness, and minimal impact on enzyme structure. However, it faces challenges like enzyme leaching and reduced stability due to weak interactions with support materials. Current strategies focus on optimizing these interactions using advanced materials like hybrid supports, magnetic nanoparticles, and graphene oxide to improve enzyme stability and loading.

Future research should develop novel supports with better interaction capabilities to enhance the process and use computational modeling and AI to predict optimal conditions. Integrating enzyme engineering can improve stability and customize enzymes for specific industrial processes. In summary, while physical adsorption is promising, addressing its challenges with advanced materials and methodologies will lead to more efficient, stable, and versatile immobilized enzymes for sustainable industrial applications.
